# Metabolism-based isolation of invasive glioblastoma cells with specific gene signatures and tumorigenic potential

**DOI:** 10.1093/noajnl/vdaa087

**Published:** 2020-07-13

**Authors:** Stuart James Smith, Jonathan Rowlinson, Maria Estevez-Cebrero, David Onion, Alison Ritchie, Phil Clarke, Katie Wood, Mohammed Diksin, Anbarasu Lourdusamy, Richard Guy Grundy, Ruman Rahman

**Affiliations:** 1 Children’s Brain Tumour Research Centre, School of Medicine, University of Nottingham, Nottingham, UK; 2 School of Life Sciences, University of Nottingham, Nottingham, UK; 3 Division of Cancer and Stem Cells, School of Medicine, University of Nottingham, Nottingham, UK

**Keywords:** 5-aminolevulinic acid, gene expression, glioblastoma, heterogeneity, neurosurgery

## Abstract

**Background:**

Glioblastoma (GBM) is a highly aggressive brain tumor with rapid subclonal diversification, harboring molecular abnormalities that vary temporospatially, a contributor to therapy resistance. Fluorescence-guided neurosurgical resection utilizes the administration of 5-aminolevulinic acid (5-ALA) generating individually fluorescent tumor cells within a background population of non-neoplastic cells in the invasive tumor region. The aim of the study was to specifically isolate and interrogate the invasive GBM cell population using a novel 5-ALA-based method.

**Methods:**

We have isolated the critical invasive GBM cell population by developing 5-ALA-based metabolic fluorescence-activated cell sorting. This allows purification and study of invasive cells from GBM without an overwhelming background “normal brain” signal to confound data. The population was studied using RNAseq, real-time PCR, and immunohistochemistry, with gene targets functionally interrogated on proliferation and migration assays using siRNA knockdown and known drug inhibitors.

**Results:**

RNAseq analysis identifies specific genes such as *SERPINE1* which is highly expressed in invasive GBM cells but at low levels in the surrounding normal brain parenchyma. siRNA knockdown and pharmacological inhibition with specific inhibitors of *SERPINE1* reduced the capacity of GBM cells to invade in an in vitro assay. Rodent xenografts of 5-ALA-positive cells were established and serially transplanted, confirming tumorigenicity of the fluorescent patient-derived cells but not the 5-ALA-negative cells.

**Conclusions:**

Identification of unique molecular features in the invasive GBM population offers hope for developing more efficacious targeted therapies compared to targeting the tumor core and for isolating tumor subpopulations based upon intrinsic metabolic properties.

**Key Points**

- Clinical 5-ALA fluorescence can be used to isolate invasive GBM cells from the normal brain parenchyma.
- Invasive GBM cells have unique molecular targets compared to cells from the bulk tumor.
- These unique invasive targets are potentially targetable to limit invasion by GBM.

**Importance of the Study**

Targeted therapy for glioblastoma (GBM) is complicated by intratumor heterogeneity, with most attempts to treat postsurgical residual disease based on extrapolation from targets identified in the tumor core. Attempts to analyze invasive disease have been complicated by an overwhelming normal brain signal. This study uses 5-aminolevulinic acid (5-ALA) to facilitate the isolation of invasive GBM cells and interrogate them with RNAseq. We demonstrate novel molecular targets unique to the invasive population and the tumorigenicity of the 5-ALA-positive cells. We show how targeting *SERPINE1* with siRNA or pharmacological inhibitors can reduce the capacity of GBM cells to invade, giving hope that 5-ALA-based isolation may become a basis for identifying clinically relevant molecular targets on invasive GBM cells.

Overall survival for the high-grade malignant brain tumor glioblastoma (GBM) has remained disappointingly static over the last decade with a median survival of 14.6 months in patients treated radically with surgery, radiotherapy, and temozolomide.^1^ Multiple phase III trials of targeted agents based on biological data have failed to show any overall survival benefit.^2–4^ The reasons for these setbacks are complex, including potential failure to achieve sufficient concentration of agents in the tumor microenvironment, but tumor heterogeneity (both inter and intra) and hence failure to target optimal molecular candidates is also contributory.^5^ Heterogeneity in GBM is well established and variation in subclonal gene expression across tumors has been described, with truncal and discrete events developing during the spatiotemporal evolution of these tumors.^6–8^ It is now recognized that a single biopsy specimen cannot inform the broad molecular landscape of a GBM.

Tumor removed from the resection margin, where the GBM blends into and invades the normal brain, has been suggested to exhibit different genetic profiles to tumor removed from the hypoxic core or viable enhancing rim regions as defined on gadolinium contrast-enhanced MRI.^9^ Recurrence in GBM predominantly occurs in this invasive zone within 2 cm of the resection edge after surgery,^10^ and it is logical that tumor genetic profiles from this region are more likely to identify molecular targets to delay recurrence. However, biopsies taken from the invasive zone will contain substantial amounts of non-neoplastic cells, including immune infiltrates and normal CNS cells, which may dominate attempts at genome-wide analysis of the tumor component and tumor signatures of invasion.^11^

A phase III randomized clinical trial has demonstrated an increase in rates of complete resection of enhancing disease from 36% to 65% of GBM patients by the use of 5-aminolevulinic acid (5-ALA) as a surgical adjunct.^12^ 5-ALA is a porphyrin, metabolized by cells where the heme synthesis pathway is active (eg, GBM cells, but not non-neoplastic CNS cells), to the fluorescent metabolite protoporphyrin IX (PpIX; Figure 1).

**Figure 1.**
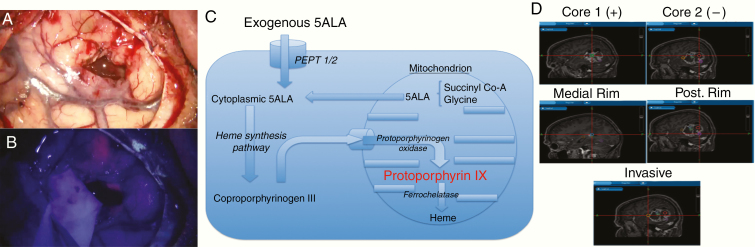
An overview of 5-aminolevulinic acid (5-ALA) (Gliolan) guided surgery and sampling technique: (A) conventional white light view through the operating microscope of temporal lobe with partially resected GBM; (B) the same view under blue light demonstrating areas of 5-ALA-induced pink tumor fluorescence; (C) metabolic pathway for fluorescent protoporphyrin IX synthesis in GBM cells after exogenous administration of 5-ALA; and (D) representative image of multiregion surgical sampling from a GBM indicating typical sample locations.

PpIX subsequently undergoes intracellular accumulation in GBM cells owing to their lack of ferrochelatase activity, with maximal excitement induced by blue light at 400–410 nm and the main emission light peaks at 635 and 704 nm (pink; Figure 1).

Areas of pink fluorescence as observed by the operating surgeon correspond to areas of high viable tumor cell density and are a suitable target for resection.^13^ The necrotic core does not fluoresce due to the lack of viable cellular metabolism, and at the peripheral invasive edge of the visible tumor, the pink fluorescence fades to blue as the tumor cell density falls. Although non- or weakly fluorescent, this region has been shown to still contain detectable numbers of invading GBM cells.^13,14^ On an individual cell basis, GBM cells in the peripheral tumor still accumulate PpIX and hence fluoresce even in the absence of fluorescence perceptible to the human eye,^15^ and we have sought to use this characteristic to isolate GBM cells from the invasive region from among the non-neoplastic parenchyma.

In this study, 58 regions from 11 patients undergoing surgery for MRI suspected GBM were taken from core areas (central 5-ALA nonfluorescent regions), rim areas (5-ALA fluorescent areas corresponding to gadolinium-enhancing areas), and the invasive zone (5-ALA weakly fluorescent, corresponding to nonenhancing areas beyond the rim of gadolinium uptake). A portion of the invasive zone sample was separated into 5-ALA-positive and 5-ALA-negative cells by fluorescence-activated cell sorting (FACS). This is the first oncological study to metabolically isolate and purify GBM cells representative of infiltrative residual disease, from which candidate therapy targets are likely more clinically relevant and present a methodological approach broadly applicable for solid cancers amenable to 5-ALA-induced metabolic labeling such as melanoma or breast cancer.^16,17^ We report on the RNAseq data acquired from these different tumor areas and validate a novel potential therapeutic target found on the invasive zone 5-ALA-positive cells.

## Materials and Methods

### Patient Information

This study included 11 (RNAseq) and 3 (gene expression array) newly diagnosed treatment-naïve patients diagnosed with operable, likely high-grade glioma on diagnostic and advanced MRI sequences. One further patient underwent RNAseq analysis of 9 samples (core/rim/invasive regions each with unsorted/5-ALA-positive/5-ALA-negative samples). All patients were operated on by a single surgeon at a major regional Neuroscience center, with specimens collected after informed consent was obtained for this purpose from the patients and under ethics committee approval (11/EM/0076).

### Surgical Sampling

5-ALA was administered orally to patients 2–4 h prior to surgery at 20 mg/kg dose. All patients underwent craniotomy with intraoperative image guidance and visualization of 5-ALA-induced fluorescence using an appropriately equipped operative microscope (Leica OH-X). Multiregion sampling was conducted, and the sample location determined using image guidance. Samples were taken from nonfluorescent or minimally fluorescent tumor core (corresponding to T1 with gadolinium nonenhancing or heterogeneously enhancing central tumor), viable fluorescent tumor rim (corresponding to peripheral strongly gadolinium-enhanced areas on T1 MRI), and from the areas of 5-ALA-induced fluorescence furthest into the area of MRI T2 high signal beyond the bulk tumor, where tumor blended into the brain in an invasive fashion (nonenhancing on T1 with gadolinium; Figure 1D). Histological diagnosis of GBM was confirmed by intraoperative smear and formal postoperative diagnosis (including IDH mutations, ATRX mutation, and MGMT methylation status) by the regional brain tumor neuropathology service (Supplementary Table 1).

### Fluorescence-Activated Cell Sorting

Cells were sorted using the MoFlo XPD sorter (Beckman Coulter) with excitation at 405 nm and emission detection at 605–625 nm. For increased purity, cells were sorted twice consecutively. U251 GBM cells incubated for 2 h with and without 5-ALA were used as controls to set gates for sorting. The positive and negative sorted cells were centrifuged at 800 rpm (180 × *g*) for 5 min at room temperature and cell pellets snap frozen.

### Cell Lines and Primary Cultures

All cell lines used in this project (Supplementary Table 2), except C17.2, were of human origin. C17.2 cells are multipotent neuronal stem cells derived from mouse cerebellum. U251 cells (confirmed on Short Tandem Repeat (STR) genotyping; Supplementary Figure 1), isolated from the core of a GBM tumor and sourced commercially, were used as a biological positive control for GBM cells. The Glioma INvasive margin (GIN) cell lines, isolated from the tumor infiltrative edge, were derived in-house from surgeries at the Queen’s Medical Centre, Nottingham (comparable to their respective primary tissue on STR; Supplementary Figure 1).

### Other Methods

Full description of methods is provided in Supplementary Methods but in brief, cell culture was undertaken with/without 1 mL 20 mM 5-ALA (Sigma-Aldrich) per 75 mL flask. Presto blue (Invitrogen) was performed according to the manufacturer’s protocol. Real-time PCR was performed with iQ SYBR Green Supermix 2x (Bio-Rad) and immunohistochemistry with DAKO reagents and secondary antibody. Transcriptomic libraries were prepared using the NEBNext Poly(A) mRNA Magnetic Isolation Module (NEB: E7490), the NEBNext Ultra Directional Library Kit for Illumina (NEB: E7420), and the NEBNext Multiplex Oligos for Illumina (Index Primers Set 1; NEB: E7335L). Raw data have been deposited at ArrayExpress, accession number E-MTAB-8743. siRNA knockdown was performed using 25 nM of *SERPINE1* targeting and nontargeting sequences (Dharmacon). Invasion assays were conducted using 24-well ThinCert cell culture inserts (Greiner) coated with 10 μg of collagen IV (Cultrex, Trevigen). In vivo patient-derived xenografts were performed in male Rag2/Il2rg (RAG) immunodeficient mice (10–12 weeks old) according to National Cancer Research Institute and Laboratory Animal Science Association guidelines.

## Results

### Patient Demographics

The RNAseq patient cohort (*n* = 11; Supplementary Table 1) had a median age of 58.7 years and consisted of 5 males and 6 females. All patients had neuropathologically confirmed GBM with an expression of wild-type isocitrate dehydrogenase 1 (IDH-1) and wild-type ATRX and underwent resection of their tumor with 5-ALA guidance. All patients had multiregion sampling successfully performed to obtain samples from core, rim, and invasive tumor regions. Eight patients received 60 Gy radiotherapy in 30 fractions with concomitant and adjuvant temozolomide, 2 patients received a radiotherapy regime only, and 1 patient received best supportive care (patient choice). Median survival was 15.0 months.

### In Vitro Optimization of 5-ALA FACS Parameters

**Figure 2.**
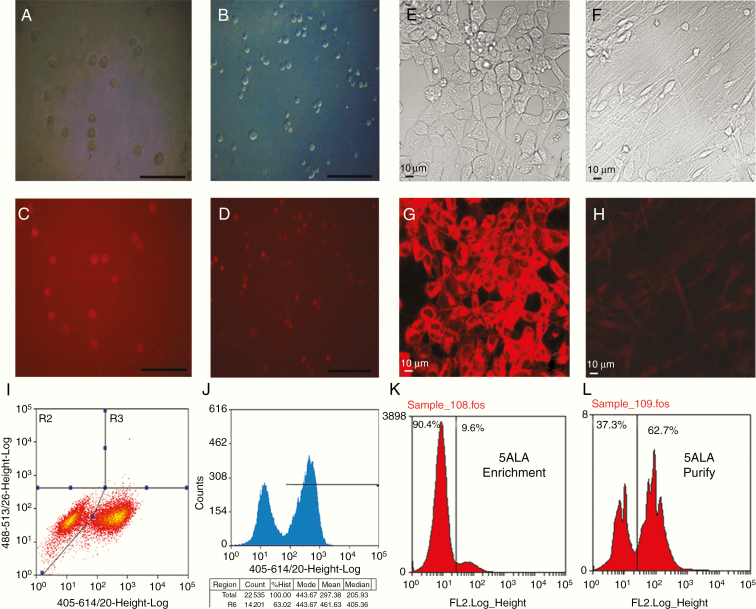
Administration of 5-ALA, to cell lines and in fluorescent-activated cell sorting (FACS): (A and C) U251 GBM cells imaged with bright field microscopy (A) and fluorescence microscopy after administration of 5-ALA (C) showing fluorescence in the vast majority of cells (scale bar 50 μm). (B and D) GIN17 primary low-passage GBM cells imaged with bright field microscopy (B) and fluorescence microscopy (D) after administration of 5-ALA with near-universal fluorescence (scale bar 50 μm). (E–H) Bright-field (E and F) and fluorescence (G and H) microscopy of human neural cell stem cells in an undifferentiated state (E and G) and after differentiation to astrocytes (F and H) with significantly reduced 5-ALA-induced fluorescence in their differentiated state. (I and J) FACS profile of a 50:50 mixture of 5-ALA-treated/untreated U251 cells demonstrating clear separation of unexposed cells (left-hand peak) from cells exposed to 5-ALA (right-hand peak). (K and L) Primary tumor specimens from patients undergoing isolation for 5-ALA fluorescence positive tumor cells, demonstrating first stage enrichment, followed by purification on the second sort.

5-ALA was applied to established cell lines and primary low-passage GBM cultures (Glioma INvasive lines—GIN) and the percentage of positive fluorescent cells assessed using bright field and fluorescent microscopy (Figure 2A–D).

The percentage of 5-ALA-induced fluorescent cells was 84.2% (±6.8%) in U251 commercial GBM cells and ranged from 83.6% (±5.1%) to 78.8% in primary low-passage GIN cell lines. One cell line derived from a recurrent GBM showed auto-fluorescence (44.4% of cells) but all other cell lines derived from primary resections had less than 5% fluorescence when studied without 5-ALA (*P* ≤ .05). The neural stem cell line C17.2 (after differentiation) demonstrated 1.5% (±0.9%) fluorescent cells with 5-ALA and 0% fluorescence without 5-ALA, significantly fewer than the actively dividing tumor cell lines (*t*-test *P* value ≤ .001). Similar results were obtained with the H1 human neural stem cell line, which exhibited fluorescence in its undifferentiated stem cell state but markedly reduced fluorescence after differentiation to post-mitotic astrocytes (*P* ≤ .01; Figure 2E–H). Stem cell lines post-differentiation demonstrated no difference between fluorescence levels in 5-ALA exposed and untreated cells (*P* ≥ .05).

Scatter graphs of FACS data were created for each 5-ALA-positive and negative sample from each cell line with the *x*-axis representing the size and the side scatter on the *y*-axis representing granularity. A gate was created based on a U251 sample and applied to the remaining samples. The cells within the gate were analyzed, using the FL11 channel to identify fluorescence levels. Fluorescence intensity levels varied within tumor cell populations, whether exposed to 5-ALA or not, but the clear separation between 5-ALA-positive and negative cells was apparent (Figure 2I). Percentage of cells demonstrating high levels of fluorescence on FACS after application of 5-ALA to each cell line was highly concordant with bright field microscopy (*R*^2^ = 0.98 and *P* ≤ .001; Supplementary Figure 2A). Gating and separation of cells into fluorescence positive and negative populations was performed using FACS via a 2-stage process involving the enrichment of the 5-ALA-positive population, followed by purification of the enriched population (Figure 2J–L). No significant cell viability differences were observed between 5-ALA exposed and unexposed cells for any cell lines (all *t*-test *P* values > .05; Supplementary Figure 2).

### Isolation and RNAseq Analysis of Different Tumor Regions

**Figure 3.**
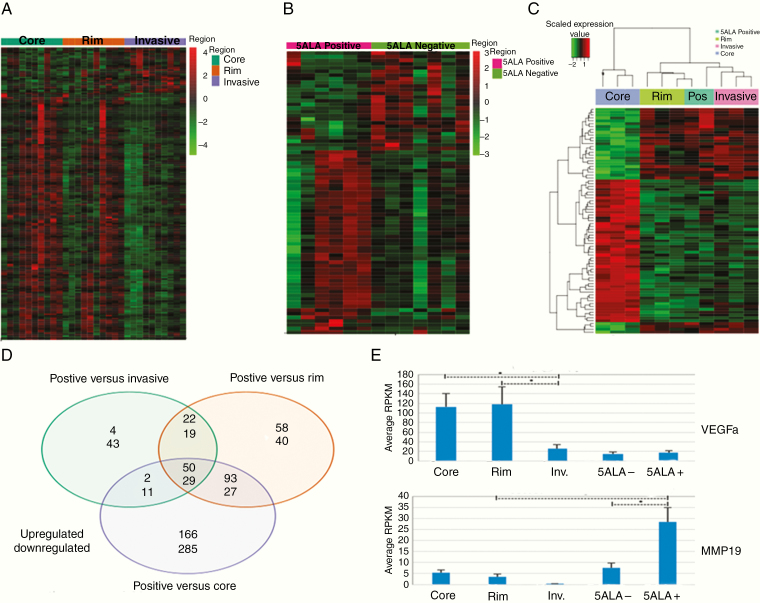
RNAseq bioinformatics results. (A) Heat map of RNAseq data demonstrating differentially expressed genes (*P* < .05 after multiple test correction) patterns between core/rim and invasive margin regions. (B) Heatmap of RNAseq data showing differentially expressed (*P* < .05 after multiple test correction) genes between 5-ALA-positive and 5-ALA-negative cells isolated from the invasive tumor regions. (C) Heatmap showing differentially expressed genes (*P* < .05 after multiple test correction) between 5-ALA-positive cells (Pos) and unsorted mixed tumor regions from the core, rim, and invasive margin. (D) Venn diagram of numbers of genes differentially over (top number) and under (bottom number) expressed between intratumor regions and 5-ALA-positive cells from the invasive region. (E) Examples of expression patterns of significantly differentially expressed genes of interest (*VEGFA* and *MMP19*), comparisons with an asterisk with *P* < .05, demonstrating significantly reduced expression levels of *VEGFA* in the invasive regions compared to core and rim and significantly elevated levels of *MMP19* in the invasive regions and in particular in 5-ALA-positive cells.

Using FACS and gating methodology based on in vitro parameters, we proceeded to optimize the 5-ALA fluorescence method on surgically resected GBM as a test-bed before applying the methodology to the experimental patient cohort. We were successful in gating a 5-ALA fluorescent population of cells that ranged from 0.72% to 9.6% of the total cell population in the invasive zone samples, with a mean of 1.52% (±0.22%; Supplementary Figure 2). Median time from sample resection to end of the FACS process was 146 min, enabling utilization of the persistent 5-ALA fluorescence postsurgery. RNAseq analysis on individual regions demonstrated significant differences between unsorted tumor regions (core, rim, and invasive). A median read of 106.8 million reads was achieved per unsorted sample, median 69.7 million reads per FACS sorted sample and with 5 samples excluded due to poor read quality. About 2567 genes were significantly differentially expressed between core tumor and unsorted invasive region samples (full gene list in Dataset 1) with clear separation of different microenvironmental regions when analyzed as a heatmap for significantly differentially expressed genes (Figure 3A), with pathway analysis suggesting many of these were, as would be predicted, associated with a normal brain, an obstacle which has historically obscured GBM invasive region biological information.

Many over-represented signaling pathways (Supplementary Table 3) in the group of genes upregulated in invasive regions were associated with synaptic activity and other CNS processes (eg, neuroactive ligand–receptor interaction, GABAergic synapses, glutaminergic synapses, and serotonergic synapses).

### Comparison of 5-ALA/FACS-Positive and Negative Invasive Cell Populations by RNAseq Analysis

A comparison was made by RNAseq between 5-ALA-positive and negative cells isolated from the GBM tumor invasive margin. Sufficient good quality reads to allow comparison were obtained for six 5-ALA-positive and seven 5-ALA-negative tumor regions. Seventy-eight genes were significantly differentially expressed with separation of fluorescent and nonfluorescent cell populations on a heatmap (Figure 3B) of the differentially expressed genes. Pathway analysis revealed significant enrichment for pathways associated with cellular motility and invasion, with enrichment for immune responses in addition (Supplementary Table 4 with full gene list in Supplementary Table 5). Seventy-eight genes were significantly upregulated between 5-ALA/FACS-positive cells and the unsorted invasive region, with 102 genes significantly downregulated. Similar differences were observed between 5-ALA-positive cells and both core and rim regions, with the greatest number of genes differing between 5-ALA-positive invasive cells and unsorted tumor core regions (Figure 3D). The 79 genes (50 upregulated and 29 downregulated) that were significantly different between 5-ALA-positive cells and all unsorted regions are listed in Supplementary Table 9. The high number of differentially expressed genes underscores the dissimilarities between 5-ALA-positive invasive cells and tissues from the GBM core region, which have been historically the basis of the majority of genomic studies to date based on the sampling strategies described where samples are only utilized if composed of very high tumor cell purity. Cross-comparison of the differentially expressed genes between the different regions showed that only 9.1% of differentially expressed genes were conserved among the 3 regions, indicating a high degree of GBM intratumor heterogeneity based upon RNAseq expression levels. Pathway analysis showed significant differences in extracellular matrix (ECM) organization and metabolic processes (Supplementary Table 6).

**Figure 4.**
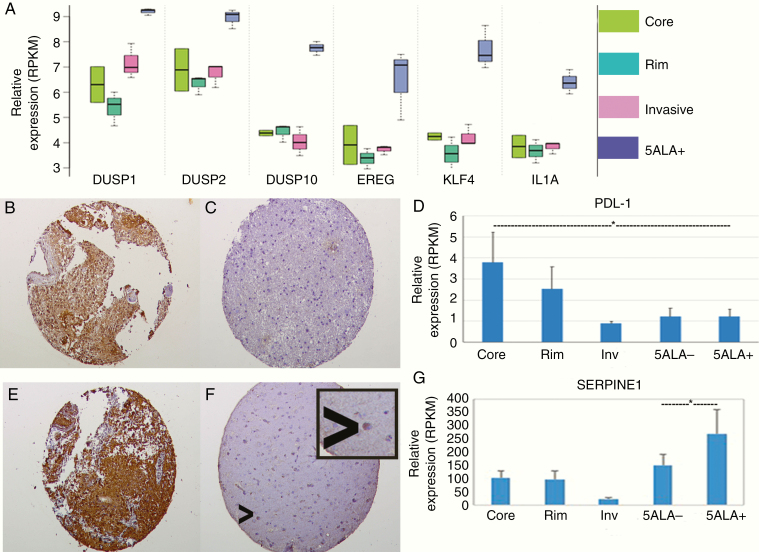
Expression levels of genes and proteins of interest in tumor and FACS isolated samples. (A) Expression profiles across tumor regions and in 5-ALA-positive cells for selected genes demonstrating expression patterns varying in the unsorted invasive region and particularly in 5-ALA-positive cells isolated by FACS. (B and C) Immunohistochemistry for VEGFA (×40 images) for a core (B) and invasive area (C) from the same GBM demonstrating representative high and low expression of VEGFA, respectively. (D) RNAseq expression profiles for the *PDL1* immune checkpoint molecule, showing high *PDL1* expression in the tumor core and low expression in 5-ALA-positive invasive cells (*t*-test *P* < .05, indicated by an asterisk). (E and F) Immunohistochemistry for SERPINE1 images (×40 images) showing high SERPINE1 expression in the tumor core (E) and overall low expression in the invasive zone (F); occasional positive cells in the invasive region (arrowhead) are hypothesized to be invasive GBM cells (magnified view of individual positive cell as an insert). (G) Expression levels on RNAseq of *SERPINE1* in the unsorted core, rim, and invasive tumor areas and 5-ALA-positive and negative invasive cells, with elevated expression in the 5-ALA-positive FACS isolated population (significance compared to 5-ALA-negative cells *t*-test *P* = .014, indicated by an asterisk).

To test the overlap between 395 upregulated genes in 5-ALA/FACS-positive invasive cells and the genotypic classifier developed by Verhaak et al.,^18^ we used the GeneOverlap R package and Fisher’s exact test. There was significant enrichment for the mesenchymal subgroup gene signature (37 of 216 GBM mesenchymal associated genes) but not for other GBM subgroup gene signatures (*P* ≤ .001, Fisher’s exact test), suggesting the invasive cells carry a predominantly mesenchymal genotype. Key oncogenes showed differing expression profiles depending on which region of the tumor was analyzed, with lower expression of *SOX2* or elevated expression of *SERPINE1* and *MMP19* (Figure 3E) in 5-ALA/FACS-positive cells, for example. For other markers, eg, *OLIG2*, there were no significant differences between 5-ALA-positive cells and unsorted cell populations. All invasive area cells (mixed unsorted, 5-ALA negative, and 5-ALA positive) showed low expression levels of *VEGFA* compared to tumor core and rim regions (Figure 3E). 5-ALA/FACS-positive cells were generally enriched for inflammatory response genes and antiapoptotic genes, with comparative downregulation of hypoxia response and angiogenic genes on pathway analysis. Overall immunophenoscores (Supplementary Figure 3E and F) were similar between regions but an expression of some conventional immune targets such as *PDL1* was found to be higher in the core but importantly, with no elevation in 5-ALA-positive invasive cells (Figure 4D).

For one further additional GBM patient, 5-ALA sorting and RNAseq analysis were performed for all 3 regions (core, rim, and invasive areas). A global correlation comparison was made for the RNAseq expression signatures between the different unsorted regions of the same tumor and 5-ALA/FACS-positive and negative cells from the respective region. 5-ALA-negative cells isolated from the invasive margin were extremely similar to the unsorted population from this region (*R* = 0.99), whereas 5-ALA-positive cells isolated from the invasive margin showed clearer differences (*R* = 0.83; Supplementary Table 7). 5-ALA/FACS-negative cells isolated from the tumor core were also similar to the unsorted tumor core population, possibly due to these representing tumor cells unable to metabolize 5-ALA to the fluorescent product due to the hypoxic conditions within this central tumor region.

Validation real-time PCR studies were undertaken for 4 genes, all of which demonstrated fold changes in the same direction and similar magnitude between 5-ALA-positive and negative cells to those suggested by the RNAseq study (Supplementary Figure 3A–C). We chose to focus on genes with high differential expression between 5-ALA-positive and negative cells and included *VEGFA* as a recognized therapeutic target.

### Gene Expression Array Analysis

To cross-validate RNAseq data and gain further insight into the molecular characteristics of the GBM invasive region and its relationships to other intratumor regions, we used microarray profiling to derive gene expression signatures representative of the invasive-enriched populations using freshly sorted cells from invasive regions prospectively from 3 additional GBM patients (Supplementary Table 1). Hierarchical clustering based on conserved genes again showed distinct clustering of the GBM samples from different regions. Significant differences in expression were also visualized between FACS isolated fluorescence positive and unsorted cells (Figure 3C). Comparison between the RNAseq dataset and the independent gene expression array dataset showed 61 genes with a significantly different expression between 5-ALA-positive and negative cells and fold change in the same direction in both transcriptomic studies (Supplementary Table 8). Gene ontology (GO) enrichment analysis showed that the 5-ALA-positive cells were enriched for GO categories related to inflammatory response, immune response, response to lipopolysaccharide, antiapoptosis, inactivation of MAPK activity, positive regulation of cell division, and negative regulation of cell proliferation. The Kyoto Encyclopedia of Genes and Genomes (KEGG) pathway analysis attributed upregulated genes to several signaling pathways such as MAPK, NOD-like receptor, chemokine signaling, toll-like receptor, and cytosolic DNA-sensing, consistent with the observed GO categories. In contrast, downregulated pathways in 5-ALA-positive cells were significantly enriched for the GO categories of response to hypoxia, cell adhesion, angiogenesis, and cell differentiation; and for the KEGG pathways of ECM–receptor interaction, pathways in cancer, focal adhesion, and protein digestion and absorption. Many genes linked to cellular invasion were identified as having unique expression profiles in 5-ALA/FACS-positive invasive cells on both RNAseq and on the gene expression microarray, such as *DUSP* family genes, *EREG* and *KLF4* (all upregulated relative to the core, rim, and invasive tissue regions). Other genes linked to immune modulation were upregulated by the 5-ALA/FACS-positive invasive population, eg, *IL1A* (Figure 4A).

Pathway analysis of differentially expressed genes showed significant enrichment for pathways such as cell migration, ECM organization, and peptidase activity, suggesting that the 5-ALA-positive population represents a molecular snapshot of true invasive GBM components (Supplementary Figure 4) when isolated as a minority population from the infiltrative edge of the tumor.

### Analysis of the Impact of FACS Processing of Samples

We wished to exclude any confounding effect by the FACS process on true in situ gene expression signatures. Therefore, for one additional patient, FACS based on 5-ALA fluorescence was conducted on all regions of the tumor (core, rim, and invasive areas) and RNAseq analysis performed. A comparison was then made to unsorted cells to establish whether any systematic changes in patterns of gene expression were attributable to the process of dissociation of tumor tissue and FACS. Systematic changes due to FACS were only detected for a small number of genes, known to be stress response genes, indicating an effect for certain genes such as *TNF-α* (Supplementary Figure 3D). Crucially, no FACS-induced effect was evident for the genes identified as differentially expressed and involved in migratory or ECM modification pathways. *TNF-α* was consistently overexpressed in all cells that had undergone FACS but interestingly was expressed at higher levels in 5-ALA-positive cells compared to 5-ALA negative, perhaps indicating a differing stress response between tumor and nontumor cells.

### Immunohistochemical Validation

Selected differentially expressed genes were also examined for expression at the protein level by immunohistochemistry. Results replicated the RNAseq findings with decreased expression of VEGFA and increased expression of SERPINE1 in tumor core tissue relative to tumor invasive margin tissue (Figure 4B/C and E/F) with scoring in Supplementary Figure 5A and B. However, individual SERPINE1-positive cells in the invasive margin may correspond to the 5-ALA-positive tumor subpopulation expressing SERPINE1 at a high level (Figure 4F), but existing as a minority population within the total unsorted tissue sample, with a predominance of low/absent SERPINE1 expression in normal brain harboring these invasive GBM cells.

### The Effect of SERPINE1 on Proliferation and Invasion

**Figure 5.**
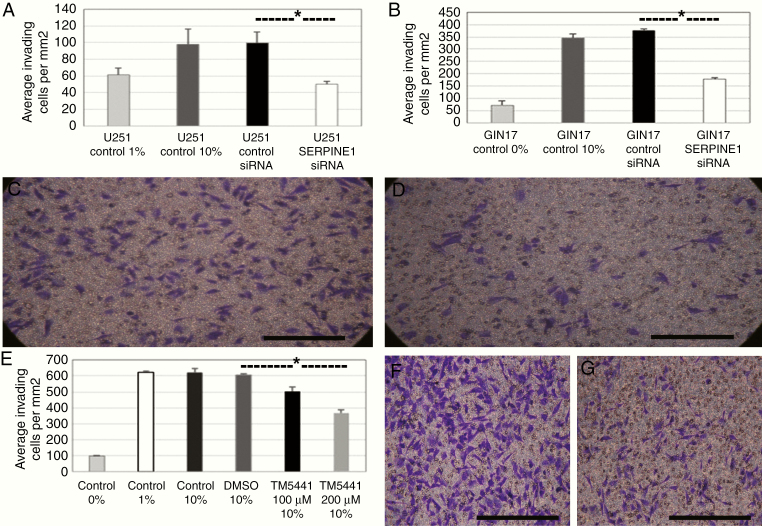
Effects on GBM invasion upon siRNA-mediated posttranscriptional inhibition of *SERPINE1* in vitro. (A and B) Average number of invading U251 (A) and GIN17 (B) GBM cells per mm^2^ after 24 h in the transwell plate in untreated controls with 0–1% FBS, 10% Fetal Bovine Serum (FBS), control siRNA transfection (10% FBS), or *SERPINE1* siRNA transfection (10% FBS; asterisk indicates significant reduction [*t*-test *P* < .05] in migrating cells between control siRNA and *SERPINE1* targeting siRNA for U251 (A) and GIN17 (B)). (C and D) ×40 images of U251 cells with control siRNA transfection (C) and *SERPINE1* siRNA transfection (D) on transwell collagen invasion assay. (E) The average number of invading GIN17 GBM cells per mm^2^ of the transwell plate upon 24 h exposure to 100 and 200 μM TM5441 (SERPINE1 inhibitor) compared to controls with 0%, 1%, and 10% serum in the lower chamber and with Dimethyl Sulfoxide control, showing significant reduction with the inhibitor (*t*-test *P* < .05, indicated by an asterisk). (F and G) ×40 images of GIN17 cells with 10% FBS control (F) or TM5441 200 μM (G) showing the number of cells visible on the transwell collagen invasion assay.

Validation of the functional effects of genes identified as associated with the infiltrative phenotype of invasive GBM cells was undertaken, using *SERPINE1* as an example of a candidate gene consistently upregulated in 5-ALA/FACS-positive cells from the invasive region compared to the unsorted mixed population from the invasive regions (*t*-test *P* = .006) and 5-ALA/FACS-negative cell populations (*t*-test *P* = .014; Figure 4G). Transient siRNA knockdown of the *SERPINE1* gene was undertaken and the effect on cellular proliferation assessed. We found no significant difference between untransfected, control (scrambled) siRNA and *SERPINE1*-specific siRNA in terms of cellular growth and proliferation of GBM cells in vitro (Supplementary Figure 5C and D). Using a transwell collagen barrier assay, we then proceeded to assess the effect of *SERPINE1* knockdown on the in vitro capacity of established and primary GBM cell lines to invade. We found that knockdown of the gene significantly (*t*-test *P* < .05) reduced the number of U251 and GIN17 GBM cells invading through the collagen barrier compared to untransfected or control siRNA cells (Figure 5A–D).

TM5441 is a potent and specific inhibitor of SERPINE1, which is bioavailable after oral administration. It has been noted to have cardioprotective effects but without the hemorrhagic complications associated with other SERPINE1 inhibitors,^19^ but has not been investigated as a possible repurposed GBM treatment. Upon administration of TM5441 to the transwell assay, a significant reduction (*t*-test *P* < .05) in the number of migrating cells was observed (Figure 5E–G) even after controlling for a reduction in cell numbers due to the antiproliferative effect of TM5441 (Supplementary Figure 5E and F). A second, less specific SERPINE1 inhibitor (Tiplaxtinin) had a similar effect on viability but no significant effect on invasion after controlling for the decrease in cell viability (Supplementary Figure 5G–I).

### Confirmation of 5-ALA/FACS-Positive Tumorigenicity In Vivo

**Figure 6.**
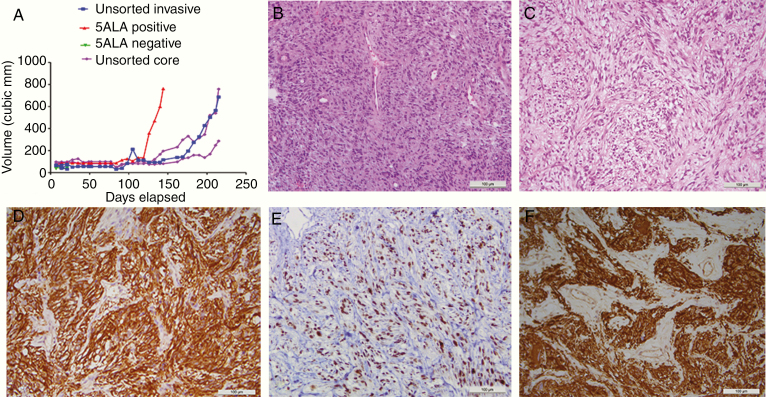
In vivo growth and immunohistochemistry. (A) Relative growth rates over time since the implantation of mixed unsorted (invasive margin or tumor core) cell xenografts (blue and purple lines), 5-ALA/FACS-negative cells (with no measurable tumor after day 20, green lines), and 5-ALA/FACS-positive invasive cells (red line), showing no growth for 5-ALA/FACS-negative cells and quicker growth for 5-ALA/FACS positive compared to unsorted mixed cells. (B) H&E of patient GBM (×40) from which a 5-ALA/FACS-positive xenograft was derived. (C) H&E of a flank xenograft in mouse, demonstrating GBM-like morphology. (D) Positive GFAP (astrocytic marker) IHC for mouse 5-ALA/FACS-positive GBM xenograft. (E) Positive IHC for Ki67 (proliferative marker) for mouse flank 5-ALA/FACS-positive GBM xenograft. (F) Positive IHC for vimentin (mesenchymal marker) in mouse 5-ALA/FACS-positive GBM xenograft (all scale bars 100 μm).

To confirm that 5-ALA-positive cells isolated from the resected GBM invasive tissue margin by FACS, represented a subpopulation enriched for tumor cells, multiple subcutaneous xenograft implants were performed using different regions from the resected primary tumor. No tumor uptake was evident in any animal injected with 5-ALA/FACS-negative cells after approximately 200 days. Animals with mixed unsorted invasive margin or core cell injections all grew visible tumors by the 200-day mark, whereas both animals injected with 5-ALA/FACS-positive cells grew tumors requiring sacrifice at 138- and 145-day postimplant, respectively (Figure 6A). 5-ALA/FACS-negative injections (green line) did not generate a subcutaneous tumor (the green line near the *x*–*y* axis intersection represents a minimal cubic volume caused by the injection of 5-ALA/FACS-negative cells, which subsequently did not engraft and proliferate). The number of animals in this study is insufficient to establish whether tumor uptake rate (and therefore tumor aggressiveness) in 5-ALA/FACS is significantly greater than the uptake rate for unsorted invasive margin and core tumor cells.

A 5-ALA/FACS-positive tumor was serially transplanted into secondary recipients on day 145, with an increased rate (approximately double) of tumor uptake evident (69 days to reach tumor diameter of 7.2 mm, relative to 119 days for a 6.8 mm diameter tumor in the primary recipient; Supplementary Figure 6D). Based on standard histological staining, the 5-ALA/FACS-positive tumor xenograft closely resembled the primary GBM from which it was derived (Figure 6B and C). Furthermore, immunohistochemical analysis of the tumor xenograft generated from 5-ALA/FACS-positive cells revealed a mitotically active (Ki67 positive) tumor, negative for the smooth muscle marker reticulin, but positive for glial fibrillary acidic protein (astrocytic marker) and vimentin (mesenchymal marker), collectively confirming glial and tumor origin of the xenograft from 5-ALA-positive GBM cells (Figure 6D–F).

## Discussion

The causes of the failure of molecularly targeted phase 3 trials and the high levels of treatment resistance exhibited by GBM are multifactorial, including innate upregulation of resistance-associated genes, failure of drugs to achieve therapeutic concentrations within the tumor microenvironment, and the capacity of GBM cells to rapidly adapt to the selection pressures. A key component of treatment resistance is the highly heterogeneous nature of individual GBMs, where diverse subclonal cell populations are present at each stage of disease progression^20^ and microenvironment.^21^ Studies have shown that heterogeneity extends to a single cell level^8^ and exists on epigenetic, genetic, and transcriptional levels^22^ with important consequences for properties such as temozolomide resistance, the first-line standard of care agent for GBM.^23^

The current study sought to characterize heterogeneity on an intraregional level and assess whether there is a consistent molecular pattern in different tumor regions. Our particular focus was the invasive GBM cells migrating into the brain parenchyma, as this tumor subpopulation most closely reflects residual disease which cannot be removed safely by surgery. We have isolated these cells using a novel 5-ALA metabolism-based method which is more inclusive of the totality of the tumor cell population(s) than previous attempts to study this population(s) based on subclonal genetic markers, eg, EGFRVIII. We present, to the best of our knowledge, the first comprehensive RNAseq study of purified invasive GBM cells isolated in situ from patients. Our findings demonstrate that there is a specific molecular signature for this cell population with a clear distinction from the normal brain. The fluorescence positive cells are also different than the bulk tumor (core and rim) that has been traditionally studied in most previous comprehensive genome-wide analyses.^24^ We show that the invasive tumor cell population is tumorigenic, recapitulating fully the histological heterogeneity of the parent GBM.

In our study, proliferation and migration/invasion were broadly dichotomous with core and rim cells displaying highly proliferative genotypes. In contrast, many of these pathways were only expressed at low levels in the purified invasive cells, with a far stronger representation of genes and pathways associated with ECM interaction, invasion, and cell motility. Our findings demonstrate support for the “grow versus go” hypothesis in situ in primary GBM, albeit based on a presumably transient molecular snapshot at a particular disease stage. Previous studies examining a limited set of markers have suggested this phenomenon occurring in GBM cells with differing sets of transcription factors activated in migrating versus migration-restricted cells.^25^ Our unsupervised analysis demonstrates the differential activation of pathways in the clinical setting. One specific target we identify is the *SERPINE1* gene (also known as plasminogen activator inhibitor 1 [*PAI-1*]). This gene is highly expressed in the 5-ALA-positive invasive GBM cell population in comparison to the 5-ALA-negative population. Previous analysis of TCGA genomic data has shown high *SERPINE1* expression in many GBMs and a correlation between expression level and overall survival.^26^*SERPINE1* expression is also significantly different between the circumscribed grade I pilocytic astrocytoma and grade IV GBM.^27^*SERPINE1* has diverse previously described roles including a possible function in immunomodulation^26^ and growth factor degradation.^28^ Here we show a non-canonical role in enhancing the invasive ability of GBM cells, but with no effect on cellular proliferation. Our findings are consistent with a previous report of *SERPINE1* involvement in the plasmin-related degradation of ECM to allow invasion^27^ and with broader relevance as a possible doxorubicin resistance mechanism in breast cancer by preventing cellular senescence.^28^ Pharmacological inhibition of *SERPINE1* in mice and heterozygosity for the null allele in humans are both associated with cellular genetic integrity.^29^ Specific pharmacological inhibitors of *SERPINE1* have been developed and used in humans for hypertension and antithrombosis. Clinical trials were discontinued due to excess bleeding events but interestingly, genetically heterozygotic human populations do not display excess hemorrhage, suggesting a controlled reduction in levels may be tolerable.^29^

In conclusion, we demonstrate a unique expression profile on RNAseq for an invasive GBM cell population isolated on the basis of 5-ALA-induced fluorescence from among the predominant non-neoplastic parenchyma in the invasive zone of GBM. This population appears to have reduced proliferation levels but exhibits significant upregulation of genes associated with invasion and migration. We suggest that future investigation of the 5-ALA/FACS-positive invasive cell population in bigger cohorts will yield further molecular markers that are specifically altered in the residual cell population that gives rise to GBM recurrence. Interrogation of this most clinically relevant population may lead to more appropriately and more effectively targeted precision therapy for patients with malignant brain tumors, with a methodological pipeline for isolation of invasive cells, potentially applicable for other solid cancers such as malignant breast tumors.

## Supplementary Material

vdaa087_suppl_Supplementary_Table_1

vdaa087_suppl_Supplementary_Table_2

vdaa087_suppl_Supplementary_Table_3

vdaa087_suppl_Supplementary_Table_4

vdaa087_suppl_Supplementary_Table_5

vdaa087_suppl_Supplementary_Table_6

vdaa087_suppl_Supplementary_Table_7

vdaa087_suppl_Supplementary_Table_8

vdaa087_suppl_Supplementary_Table_9

vdaa087_suppl_Supplementary_Figure_1

vdaa087_suppl_Supplementary_Figure_2

vdaa087_suppl_Supplementary_Figure_3

vdaa087_suppl_Supplementary_Figure_4

vdaa087_suppl_Supplementary_Figure_5

vdaa087_suppl_Supplementary_Figure_6

vdaa087_suppl_Supplementary_Figure_Legends

vdaa087_suppl_Supplementary_Methods

## Notes

**Authorship statement**. S.J.S. and R.R. conceptualized, planned the project, and wrote the manuscript. J.R. performed FACS, biostatistics (with A.L.) and performed immunohistochemistry, PCR, and invasion assays. M.E.C. performed FACS, the gene expression arrays, and cell culture work. D.O. designed, optimized, and performed FACS studies. A.R. and P.C. performed all in vivo work. K.W. studied the effects of 5-ALA on in vitro cell cultures. M.D. assisted with stem cell culture and differentiation studies. R.G.G. assisted with project planning, manuscript writing, and analysis.
