# Supplementary material for: Metabolism-based isolation of invasive glioblastoma cells with specific gene signatures and tumorigenic potential
Source: Neurooncol Adv. 2020 Jul 13;2(1):vdaa087. doi: 10.1093/noajnl/vdaa087 (PMC7462276; doi:10.1093/noajnl/vdaa087)
Supplement: vdaa087_suppl_Supplementary_Table_1 [file vdaa087_suppl_supplementary_table_1.docx]

| **Sample Number** | **Analysis** | **Cell Line Derived** | **Histology** | **IDH-1** | **ATRX** | **Date of Surgery** | **Tumour Site** | **Resection Status** | **Treatment RT/CT** | **Alive** | **Survival (months)** | **6 Month Survival** | **12 Month Survival** | **Age (years)** | **Sex** | **MGMT status (percentage methylated)** |
| --- | --- | --- | --- | --- | --- | --- | --- | --- | --- | --- | --- | --- | --- | --- | --- | --- |
| **25** | RNAseq | GIN-25 (senesced at p4) | GBM | WT | WT | 08/10/15 | Left parietal | 99% | 60/TMZ | No | 12.9 | Yes | Yes | 60 | F | 0 |
| **27** | RNAseq | GIN-27 | GBM | WT | WT | 22/10/15 | Right temporal | 99% | 60/TMZ | Yes | 25.7 | Yes | Yes | 30 | F | 75 |
| **28** | RNAseq | GIN-28 | GBM | WT | WT | 21/01/16 | Right frontal | 98% | Pall Rad | No | 3.0 | No | No | 71 | M | 0 |
| **29** | RNAseq | GIN-29 (senesced at p4) | GBM | WT | WT | 28/01/16 | Left frontal | 100% | 60/TMZ | Yes | 23.5 | Yes | Yes | 67 | F | 0 |
| **30** | RNAseq | (GIN-30) | GBM | WT | WT | 18/02/16 | Left frontal | 100% | BSC | No | 10.1 | Yes | No | 53 | M | 0 |
| **31** | RNAseq | GIN-31 | GBM | WT | WT | 09/03/16 | Right temporal | 100% | 60/TMZ | No | 17.0 | Yes | Yes | 57 | F | 0 |
| **32** | RNAseq | GIN-32 (Did not grow) | GBM | WT | WT | 28/04/16 | Right parietal | 100% | 60/TMZ | No | 18.7 | Yes | Yes | 54 | F | 25 |
| **33** | RNAseq | GIN-33(senesced at p5) | GBM | WT | WT | 26/05/16 | Right parietal | 100% | 60/TMZ | No | 11.2 | Yes | No | 70 | M | 0 |
| **34** | RNAseq | (GIN-34) | GBM | WT | WT | 13/06/16 | Left parietal | 100% | 60/TMZ | Yes | 18.1 | Yes | Yes | 33 | M | 0 |
| **37** | RNAseq | (GIN-37) | GBM | WT | WT | 20/10/16 | Right parietal | 100% | 60/TMZ | No | 11.8 | Yes | No | 63 | F | 0 |
| **58** | RNAseq | GIN-58 | GBM | WT | WT | 28/09/17 | Left frontal | 100% | Pall Rad | No | 5.3 | No | No | 78 | F | <10 |
|  |  |  |  |  |  |  |  |  |  |  |  |  |  |  |  |  |
| **2** | GE chip | GIN-27 | GBM | WT | Mut | 22/10/15 | Right temporal | 99% | 60/TMZ | Yes | 24.7 | Yes | Yes | 30 | F | 75 |
| **3** | GE chip | GCE-28 and GIN-28 | GBM | WT | WT | 21/01/16 | Right frontal | 99% | BSC | No | 3.1 | No | No | 71 | M | 0 |
| **6** | GE chip | GCE31 and GIN-31 | GBM | WT | WT | 09/03/16 | Right temporal | 100% | 60/TMZ | No | 16.1 | Yes | Yes | 57 | F | 0 |
|  |  |  |  |  |  |  |  |  |  |  |  |  |  |  |  |  |
| **Median** |  |  |  |  |  |  |  |  |  |  | 16.1 |  |  | 57 |  |  |

Supplementary Table 1: Clinical and molecular features of GBM patients included in this study
