# Supplementary material for: Metabolism-based isolation of invasive glioblastoma cells with specific gene signatures and tumorigenic potential
Source: Neurooncol Adv. 2020 Jul 13;2(1):vdaa087. doi: 10.1093/noajnl/vdaa087 (PMC7462276; doi:10.1093/noajnl/vdaa087)
Supplement: vdaa087_suppl_Supplementary_Table_2 [file vdaa087_suppl_supplementary_table_2.docx]

| **Cell line** | **Patient details** | **WHO Grade** | **Site of Disease** | **Description** | **Growth Medium** |
| --- | --- | --- | --- | --- | --- |
| U251 | Male | GBM | Cells isolated from tumour core | Primary tumour | 10% DMEM |
| GIN-3R | 54-year old female | GBM | Cells isolated from the invasive margin of a tumour in the right temporal lobe | Recurrent tumour | 10% DMEM |
| GIN-8 | 54-year old female | GBM | Cells isolated from the medial front invasive margin of a tumour in the left frontal lobe | Primary tumour | 10% DMEM |
| GIN-17 | 73-year old female | GBM | Cells isolated from the invasive margin of a tumour in the left frontal lobe via 5ALA guided surgery | Primary tumour | 15% DMEM |
| C17.2 | Mouse |  |  | Multi-potent neural stem cells from the cerebellum | C17.2 Medium |
| H1 neural stem cells | Human |  |  | Multi-potent neural stem cells |  |

Supplementary Table 2: Cell lines and primary derived cultures utilised in this study.
