# Supplementary material for: Metabolism-based isolation of invasive glioblastoma cells with specific gene signatures and tumorigenic potential
Source: Neurooncol Adv. 2020 Jul 13;2(1):vdaa087. doi: 10.1093/noajnl/vdaa087 (PMC7462276; doi:10.1093/noajnl/vdaa087)
Supplement: vdaa087_suppl_Supplementary_Table_3 [file vdaa087_suppl_supplementary_table_3.docx]

| Term ID | Pathway term | Number of genes | p-value |
| --- | --- | --- | --- |
| KEGG:04080 | Neuroactive ligand-receptor interaction | 28 | 1.04E-10 |
| KEGG:04727 | GABAergic synapse | 15 | 1.69E-08 |
| KEGG:05033 | Nicotine addiction | 11 | 1.82E-08 |
| KEGG:05032 | Morphine addiction | 15 | 2.79E-08 |
| KEGG:04723 | Retrograde endocannabinoid signaling | 15 | 0.0000263 |
| KEGG:04724 | Glutamatergic synapse | 13 | 0.0000469 |
| KEGG:04020 | Calcium signaling pathway | 16 | 0.0000691 |
| KEGG:04261 | Adrenergic signaling in cardiomyocytes | 14 | 0.000121 |
| KEGG:04726 | Serotonergic synapse | 12 | 0.000256 |
| KEGG:04721 | Synaptic vesicle cycle | 9 | 0.000436 |
| KEGG:04024 | cAMP signaling pathway | 15 | 0.0012 |
| KEGG:04725 | Cholinergic synapse | 11 | 0.00185 |
| KEGG:04921 | Oxytocin signaling pathway | 12 | 0.00581 |

Supplementary Table 3: Significantly differentially involved KEGG pathways comparing unsorted invasive region cells versus core GBM tumour cells
