# Supplementary material for: Metabolism-based isolation of invasive glioblastoma cells with specific gene signatures and tumorigenic potential
Source: Neurooncol Adv. 2020 Jul 13;2(1):vdaa087. doi: 10.1093/noajnl/vdaa087 (PMC7462276; doi:10.1093/noajnl/vdaa087)
Supplement: vdaa087_suppl_Supplementary_Table_4 [file vdaa087_suppl_supplementary_table_4.docx]

|  | **Pathway Description** | **Number of Genes** | **P-value** |
| --- | --- | --- | --- |
| 1 | Signal transduction | 34 | 1.57E-10 |
| 2 | Regulation of cell activation | 29 | 2.63E-10 |
| 3 | Positive regulation of cell activation | 21 | 1.06E-09 |
| 4 | Cell migration | 36 | 1.01E-08 |
| 5 | Positive regulation of leukocyte activation | 21 | 1.40E-08 |
| 6 | Regulation of localization | 19 | 1.54E-08 |
| 7 | Locomotion | 28 | 2.09E-08 |
| 8 | Regulation of cell proliferation | 18 | 2.33E-08 |
| 9 | Positive regulation of leukocyte proliferation | 20 | 2.39E-08 |
| 10 | Regulation of molecular function | 17 | 3.00E-08 |
| 11 | Positive regulation of lymphocyte proliferation | 17 | 3.04E-08 |
| 12 | Regulation of leukocyte activation | 19 | 3.14E-08 |
| 13 | Positive regulation of lymphocyte activation | 22 | 3.17E-08 |
| 14 | Regulation of lymphocyte proliferation | 23 | 3.24E-08 |
| 15 | Leukocyte migration | 21 | 3.43E-08 |
| 16 | Positive regulation of monocyte proliferation | 17 | 4.24E-08 |
| 17 | Regulation of monocyte proliferation | 13 | 4.32E-08 |
| 18 | Regulation of B cell activation | 19 | 6.18E-08 |
| 19 | Secretion | 22 | 6.85E-08 |
| 20 | Secretion by cell | 19 | 7.04E-08 |

Supplementary Table 4: Pathways differentially represented in 5ALA positive and 5ALA negative cells from invasive tumour regions.
