# Supplementary material for: Metabolism-based isolation of invasive glioblastoma cells with specific gene signatures and tumorigenic potential
Source: Neurooncol Adv. 2020 Jul 13;2(1):vdaa087. doi: 10.1093/noajnl/vdaa087 (PMC7462276; doi:10.1093/noajnl/vdaa087)
Supplement: vdaa087_suppl_Supplementary_Table_5 [file vdaa087_suppl_supplementary_table_5.docx]

| EnsembleID | Gene symbol | entrez | baseMean | log2FoldChange | lfcSE | stat | pvalue | P value adjusted for FDR |
| --- | --- | --- | --- | --- | --- | --- | --- | --- |
| ENSG00000224363 | FP700111.1 | NA | 34.4835501 | 5.028033379 | 0.6354412 | 7.91266506 | 2.52E-15 | 5.34E-11 |
| ENSG00000128815 | WDFY4 | NA | 6915.20496 | 1.803282714 | 0.31641953 | 5.69902463 | 1.20E-08 | 8.51E-05 |
| ENSG00000179331 | RAB39A | 54734 | 312.665155 | 1.586701758 | 0.27582812 | 5.75250177 | 8.79E-09 | 8.51E-05 |
| ENSG00000259330 | INAFM2 | NA | 9929.71777 | 1.460736271 | 0.2661016 | 5.4893931 | 4.03E-08 | 0.000213677 |
| ENSG00000107099 | DOCK8 | 81704 | 10324.942 | 1.464556894 | 0.28730092 | 5.09764083 | 3.44E-07 | 0.001457639 |
| ENSG00000099337 | KCNK6 | 9424 | 1182.05804 | 1.572215249 | 0.31520851 | 4.98785785 | 6.11E-07 | 0.001899363 |
| ENSG00000128040 | SPINK2 | 6691 | 15.2962218 | -3.203051893 | 0.64620673 | -4.9566985 | 7.17E-07 | 0.001899363 |
| ENSG00000142185 | TRPM2 | 7226 | 6668.18024 | 1.657465506 | 0.33364856 | 4.96769864 | 6.78E-07 | 0.001899363 |
| ENSG00000196542 | SPTSSB | 165679 | 134.819254 | -2.405726179 | 0.49110065 | -4.8986419 | 9.65E-07 | 0.002272285 |
| ENSG00000117519 | CNN3 | 1266 | 11135.9276 | -1.457576944 | 0.29884747 | -4.8773274 | 1.08E-06 | 0.002278837 |
| ENSG00000066294 | CD84 | 8832 | 10409.1194 | 1.300713989 | 0.26898732 | 4.83559585 | 1.33E-06 | 0.002498051 |
| ENSG00000104951 | IL4I1 | 259307 | 1714.44811 | 2.172703223 | 0.45049286 | 4.82294713 | 1.41E-06 | 0.002498051 |
| ENSG00000100647 | SUSD6 | 9766 | 13618.9522 | 1.045918484 | 0.22263555 | 4.69789513 | 2.63E-06 | 0.004284963 |
| ENSG00000181191 | PJA1 | 64219 | 1397.29543 | -1.928657869 | 0.41866035 | -4.6067364 | 4.09E-06 | 0.006191667 |
| ENSG00000150687 | PRSS23 | 11098 | 1896.33886 | -2.377466906 | 0.52377413 | -4.5391072 | 5.65E-06 | 0.00798132 |
| ENSG00000241839 | PLEKHO2 | 80301 | 10086.2995 | 1.12170508 | 0.24886721 | 4.5072434 | 6.57E-06 | 0.008698691 |
| ENSG00000141524 | TMC6 | 11322 | 9275.06992 | 1.355332977 | 0.30419072 | 4.45553696 | 8.37E-06 | 0.010431884 |
| ENSG00000196139 | AKR1C3 | 8644 | 569.670003 | -2.274545276 | 0.51612351 | -4.4069787 | 1.05E-05 | 0.011993176 |
| ENSG00000169756 | LIMS1 | 3987 | 5379.04465 | 1.507522832 | 0.34420721 | 4.37969566 | 1.19E-05 | 0.011993176 |
| ENSG00000101194 | SLC17A9 | 63910 | 1942.74787 | 1.471972675 | 0.33588697 | 4.38234525 | 1.17E-05 | 0.011993176 |
| ENSG00000184988 | TMEM106A | 113277 | 1622.73729 | 1.987312663 | 0.45356424 | 4.38154616 | 1.18E-05 | 0.011993176 |
| ENSG00000101134 | DOK5 | 55816 | 599.02325 | -1.7549551 | 0.40417361 | -4.3420824 | 1.41E-05 | 0.0131103 |
| ENSG00000229512 | AC068580.1 | NA | 152.055683 | 1.605769021 | 0.37010891 | 4.33863919 | 1.43E-05 | 0.0131103 |
| ENSG00000131196 | NFATC1 | 4772 | 1484.06729 | 1.253644902 | 0.28975204 | 4.32661279 | 1.51E-05 | 0.0131103 |
| ENSG00000111252 | SH2B3 | 10019 | 13264.6259 | 1.420081695 | 0.32857481 | 4.32194328 | 1.55E-05 | 0.0131103 |
| ENSG00000173114 | LRRN3 | 54674 | 1416.82846 | -1.846770749 | 0.43231021 | -4.2718648 | 1.94E-05 | 0.015214689 |
| ENSG00000243279 | PRAF2 | 11230 | 1978.19255 | -0.772396745 | 0.18074729 | -4.2733517 | 1.93E-05 | 0.015214689 |
| ENSG00000120457 | KCNJ5 | 3762 | 1766.03642 | 1.555450866 | 0.36780108 | 4.22905459 | 2.35E-05 | 0.016672004 |
| ENSG00000100427 | MLC1 | 23209 | 5063.28131 | -2.039442496 | 0.48239145 | -4.227775 | 2.36E-05 | 0.016672004 |
| ENSG00000132639 | SNAP25 | 6616 | 1250.15658 | -1.634759417 | 0.38603696 | -4.2347225 | 2.29E-05 | 0.016672004 |
| ENSG00000185298 | CCDC137 | 339230 | 796.635848 | 1.401613819 | 0.33308972 | 4.20791681 | 2.58E-05 | 0.017619146 |
| ENSG00000248405 | ARHGAP8 | 23779 | 70.8567432 | 1.782622165 | 0.42946057 | 4.15084013 | 3.31E-05 | 0.018593513 |
| ENSG00000166780 | C16orf45 | 89927 | 3279.90388 | -1.194817298 | 0.28846138 | -4.1420355 | 3.44E-05 | 0.018593513 |
| ENSG00000248905 | FMN1 | 342184 | 2489.71251 | 1.549049025 | 0.36996713 | 4.18699096 | 2.83E-05 | 0.018593513 |
| ENSG00000111716 | LDHB | 3945 | 27877.5212 | -0.799627189 | 0.19356137 | -4.13113 | 3.61E-05 | 0.018593513 |
| ENSG00000240720 | LRRD1 | 401387 | 802.738414 | 2.825615254 | 0.67939726 | 4.15900303 | 3.20E-05 | 0.018593513 |
| ENSG00000274210 | U1 | NA | 200.325701 | 1.557806045 | 0.37515001 | 4.15248837 | 3.29E-05 | 0.018593513 |
| ENSG00000254704 | AP002358.2 | NA | 65.8778252 | 1.880726646 | 0.45483328 | 4.13498026 | 3.55E-05 | 0.018593513 |
| ENSG00000113494 | PRLR | 5618 | 256.656284 | 2.734223985 | 0.65563001 | 4.1703765 | 3.04E-05 | 0.018593513 |
| ENSG00000207205 | RNVU1-15 | 101954267 | 218.916755 | 1.909568009 | 0.46276957 | 4.12639065 | 3.69E-05 | 0.018593513 |
| ENSG00000167703 | SLC43A2 | 124935 | 9376.05855 | 1.292517657 | 0.31267361 | 4.13376 | 3.57E-05 | 0.018593513 |
| ENSG00000281453 | TGFB2-OT1 | 103611157 | 151.460634 | -2.737711451 | 0.65526828 | -4.1780009 | 2.94E-05 | 0.018593513 |
| ENSG00000241484 | ARHGAP8 | 23779 | 82.1140759 | 1.854124144 | 0.45333854 | 4.08993272 | 4.31E-05 | 0.020422777 |
| ENSG00000128512 | DOCK4 | 9732 | 15023.0685 | 1.250285784 | 0.30616732 | 4.08366828 | 4.43E-05 | 0.020422777 |
| ENSG00000248698 | LINC01085 | 152742 | 19.5488055 | -2.191804211 | 0.53579086 | -4.0907831 | 4.30E-05 | 0.020422777 |
| ENSG00000197696 | NMB | 4828 | 748.651483 | -1.541451733 | 0.37718399 | -4.0867369 | 4.37E-05 | 0.020422777 |
| ENSG00000167202 | TBC1D2B | 23102 | 4786.47785 | 1.072077477 | 0.26345235 | 4.06934103 | 4.71E-05 | 0.021257964 |
| ENSG00000171914 | TLN2 | 83660 | 5873.74842 | 1.651472399 | 0.4065819 | 4.06184435 | 4.87E-05 | 0.021495106 |
| ENSG00000125730 | C3 | 718 | 173185.743 | 1.604441899 | 0.39626068 | 4.0489556 | 5.14E-05 | 0.021805171 |
| ENSG00000131398 | KCNC3 | 3748 | 911.38218 | 1.444337155 | 0.35638059 | 4.05279405 | 5.06E-05 | 0.021805171 |
| ENSG00000173442 | EHBP1L1 | 254102 | 7069.18297 | 1.273799222 | 0.31569189 | 4.03494439 | 5.46E-05 | 0.022257808 |
| ENSG00000259520 | LOC101928414 | 101928414 | 37.6674215 | -2.156931715 | 0.53397857 | -4.03936 | 5.36E-05 | 0.022257808 |
| ENSG00000198721 | ECI2 | 10455 | 3109.51281 | -0.826613326 | 0.20535677 | -4.0252548 | 5.69E-05 | 0.022756868 |
| ENSG00000175183 | CSRP2 | 1466 | 2659.3897 | -1.636847515 | 0.4075334 | -4.0164745 | 5.91E-05 | 0.023183754 |
| ENSG00000179583 | CIITA | 4261 | 7570.91228 | 1.743934342 | 0.43523626 | 4.00686821 | 6.15E-05 | 0.023707749 |
| ENSG00000121281 | ADCY7 | 113 | 5814.17676 | 1.493677358 | 0.37542253 | 3.97865674 | 6.93E-05 | 0.026227263 |
| ENSG00000163431 | LMOD1 | 25802 | 419.632215 | -2.229855106 | 0.56316617 | -3.9594976 | 7.51E-05 | 0.027924212 |
| ENSG00000103365 | GGA2 | 23062 | 7293.47445 | 0.712541251 | 0.18025227 | 3.95302228 | 7.72E-05 | 0.028188184 |
| ENSG00000196782 | MAML3 | 55534 | 1571.96737 | 1.185426755 | 0.30039768 | 3.94619141 | 7.94E-05 | 0.028188184 |
| ENSG00000113140 | SPARC | 6678 | 82474.6544 | -1.412355087 | 0.35801361 | -3.9449759 | 7.98E-05 | 0.028188184 |
| ENSG00000182628 | SKA2 | 348235 | 2784.04763 | -0.883008408 | 0.22446457 | -3.9338431 | 8.36E-05 | 0.029042894 |
| ENSG00000265975 | AC002091.1 | NA | 114.75643 | 1.484709974 | 0.37832261 | 3.92445479 | 8.69E-05 | 0.029711997 |
| ENSG00000255197 | AC090559.1 | NA | 775.572947 | 1.372985075 | 0.35149094 | 3.90617484 | 9.38E-05 | 0.031541997 |
| ENSG00000236360 | AL445183.3 | NA | 25.3389708 | -2.069432617 | 0.5316806 | -3.8922477 | 9.93E-05 | 0.032887255 |
| ENSG00000105339 | DENND3 | 22898 | 15399.6367 | 1.316706862 | 0.33895845 | 3.88456717 | 0.00010251 | 0.03342218 |
| ENSG00000176165 | FOXG1 | 2290 | 1980.35178 | -1.63430048 | 0.42116578 | -3.8804209 | 0.00010428 | 0.033482033 |
| ENSG00000134516 | DOCK2 | 1794 | 9921.017 | 1.199327646 | 0.31037555 | 3.86411766 | 0.00011149 | 0.034731883 |
| ENSG00000110324 | IL10RA | 3587 | 10910.473 | 0.965774309 | 0.25015838 | 3.86065145 | 0.00011309 | 0.034731883 |
| ENSG00000006047 | YBX2 | 51087 | 29.2909436 | -2.313040195 | 0.59861902 | -3.8639604 | 0.00011156 | 0.034731883 |
| ENSG00000277194 | SNORD22 | 9304 | 18.2420383 | 2.614530487 | 0.67864181 | 3.8525927 | 0.00011687 | 0.03538268 |
| ENSG00000185477 | GPRIN3 | 285513 | 4860.02892 | 1.557687937 | 0.40878964 | 3.81048779 | 0.00013869 | 0.041396889 |
| ENSG00000186635 | ARAP1 | 116985 | 17979.227 | 0.988419235 | 0.26074682 | 3.79072407 | 0.00015021 | 0.043016539 |
| ENSG00000184156 | KCNQ3 | 3786 | 3527.70807 | 1.223664442 | 0.32242111 | 3.79523672 | 0.0001475 | 0.043016539 |
| ENSG00000276269 | AL355974.3 | NA | 48.5464324 | -2.357417644 | 0.62163012 | -3.7923156 | 0.00014925 | 0.043016539 |
| ENSG00000168918 | INPP5D | 3635 | 11443.3918 | 1.327680814 | 0.35344675 | 3.75638144 | 0.00017239 | 0.048069 |
| ENSG00000125775 | SDCBP2 | 27111 | 315.825377 | -1.478674331 | 0.39363779 | -3.7564339 | 0.00017235 | 0.048069 |
| ENSG00000137841 | PLCB2 | 5330 | 12415.2644 | 1.313362551 | 0.35039492 | 3.74823515 | 0.00017808 | 0.048695771 |
| ENSG00000071246 | VASH1 | 22846 | 25940.332 | 1.35675967 | 0.36212871 | 3.74662275 | 0.00017923 | 0.048695771 |

Supplementary Table 5: Genes significantly differentially expressed between 5ALA positive and negative cells on RNAseq analysis
