# Supplementary material for: Metabolism-based isolation of invasive glioblastoma cells with specific gene signatures and tumorigenic potential
Source: Neurooncol Adv. 2020 Jul 13;2(1):vdaa087. doi: 10.1093/noajnl/vdaa087 (PMC7462276; doi:10.1093/noajnl/vdaa087)
Supplement: vdaa087_suppl_Supplementary_Table_6 [file vdaa087_suppl_supplementary_table_6.docx]

| **Pathway term** | **P-value** | **FDR q-value** |
| --- | --- | --- |
| extracellular matrix organization | 4.62E-10 | 3.91E-06 |
| extracellular structure organization | 4.62E-10 | 1.96E-06 |
| multicellular organism metabolic process | 8.80E-08 | 2.49E-04 |
| multicellular organismal macromolecule metabolic process | 9.54E-08 | 2.02E_04 |
| collagen metabolic process | 2.12E-07 | 3.60E-04 |
| multicellular organismal catabolic process | 5.72E-07 | 8.08E-04 |
| immune system process | 6.05E-07 | 7.33E-04 |
| collagen catabolic process | 8.10E-07 | 8.58E-04 |
| response to stimulus | 1.59E-06 | 1.50E-03 |
| negative regulation of endopeptidase activity | 1.73E-06 | 1.46E-03 |

Supplementary Table 6: Significantly differentially involved pathways comparing 5ALA positive invasive GBM cells vs core GBM tumour cells
