# Supplementary material for: Metabolism-based isolation of invasive glioblastoma cells with specific gene signatures and tumorigenic potential
Source: Neurooncol Adv. 2020 Jul 13;2(1):vdaa087. doi: 10.1093/noajnl/vdaa087 (PMC7462276; doi:10.1093/noajnl/vdaa087)
Supplement: vdaa087_suppl_Supplementary_Table_7 [file vdaa087_suppl_supplementary_table_7.docx]

|  | Unsorted Core | Unsorted Rim | Unsorted Invasive |
| --- | --- | --- | --- |
| Core 5ALA neg | 0.9 | 0.83 | 0.81 |
| Core 5ALA pos | 0.74 | 0.74 | 0.71 |
| Rim 5ALA neg | 0.91 | 0.79 | 0.92 |
| Rim 5ALA pos | 0.78 | 0.79 | 0.77 |
| Inv 5ALA neg | 0.78 | 0.58 | 0.99 |
| Inv 5ALA pos | 0.79 | 0.78 | 0.83 |

Supplementary Table 7: Multiple regions with comparison between unsorted and FACS sorted cells for one patient (Pearson correlation coefficents)
