# Supplementary material for: Metabolism-based isolation of invasive glioblastoma cells with specific gene signatures and tumorigenic potential
Source: Neurooncol Adv. 2020 Jul 13;2(1):vdaa087. doi: 10.1093/noajnl/vdaa087 (PMC7462276; doi:10.1093/noajnl/vdaa087)
Supplement: vdaa087_suppl_Supplementary_Table_8 [file vdaa087_suppl_supplementary_table_8.docx]

| **EnsembleID** | **Gene symbol** | **logFC_RNASEQ** | **pvalue_RNASEQ** | **padj_RNASEQ** | **logFC_ARRAY** | **pvalue_ARRAY** | **padj_ARRAY** |
| --- | --- | --- | --- | --- | --- | --- | --- |
| ENSG00000179331 | RAB39A | 1.586701758 | 8.79E-09 | 8.51E-05 | 1.49329 | 4.36E-07 | 0.0001799700 |
| ENSG00000128815 | WDFY4 | 1.803282714 | 1.20E-08 | 8.51E-05 | 1.03755 | 9.38785E-06 | 0.0049454800 |
| ENSG00000107099 | DOCK8 | 1.464556894 | 3.44E-07 | 0.001457639 | 0.707466667 | 0.000283776 | 0.0485770000 |
| ENSG00000099337 | KCNK6 | 1.572215249 | 6.11E-07 | 0.001899363 | 0.933006667 | 9.44067E-06 | 0.0049454800 |
| ENSG00000142185 | TRPM2 | 1.657465506 | 6.78E-07 | 0.001899363 | 1.007893333 | 9.88022E-05 | 0.0408670000 |
| ENSG00000128040 | SPINK2 | -3.203051893 | 7.17E-07 | 0.001899363 | -1.134953333 | 5.33286E-05 | 0.0175276000 |
| ENSG00000196542 | SPTSSB | -2.405726179 | 9.65E-07 | 0.002272285 | -1.448566667 | 4.69203E-05 | 0.0099523000 |
| ENSG00000117519 | CNN3 | -1.457576944 | 1.08E-06 | 0.002278837 | -0.98859 | 5.94098E-06 | 0.0039786000 |
| ENSG00000066294 | CD84 | 1.300713989 | 1.33E-06 | 0.002498051 | 2.155646667 | 7.89995E-06 | 0.0047276000 |
| ENSG00000100647 | SUSD6 | 1.045918484 | 2.63E-06 | 0.004284963 | 1.922376667 | 9.35821E-07 | 0.0008199700 |
| ENSG00000181191 | PJA1 | -1.928657869 | 4.09E-06 | 0.006191667 | -2.26724 | 4.8318E-07 | 0.0004516000 |
| ENSG00000150687 | PRSS23 | -2.377466906 | 5.65E-06 | 0.00798132 | -0.903296667 | 3.6983E-05 | 0.0081703400 |
| ENSG00000241839 | PLEKHO2 | 1.12170508 | 6.57E-06 | 0.008698691 | 1.27033 | 5.1175E-05 | 0.0130978650 |
| ENSG00000141524 | TMC6 | 1.355332977 | 8.37E-06 | 0.010431884 | 0.70169 | 0.00013722 | 0.0430470000 |
| ENSG00000196139 | AKR1C3 | -2.274545276 | 1.05E-05 | 0.011993176 | -1.27501 | 4.49443E-06 | 0.0028503000 |
| ENSG00000101194 | SLC17A9 | 1.471972675 | 1.17E-05 | 0.011993176 | 0.816373333 | 6.24343E-05 | 0.0219781000 |
| ENSG00000184988 | TMEM106A | 1.987312663 | 1.18E-05 | 0.011993176 | 1.36962 | 4.15606E-07 | 0.0001799700 |
| ENSG00000101134 | DOK5 | -1.7549551 | 1.41E-05 | 0.0131103 | -1.224436667 | 6.45832E-05 | 0.0264010000 |
| ENSG00000131196 | NFATC1 | 1.253644902 | 1.51E-05 | 0.0131103 | 1.782093333 | 3.80768E-08 | 0.0000085332 |
| ENSG00000111252 | SH2B3 | 1.420081695 | 1.55E-05 | 0.0131103 | 0.9803 | 9.53E-06 | 0.0059324000 |
| ENSG00000173114 | LRRN3 | -1.846770749 | 1.94E-05 | 0.015214689 | -0.879863333 | 7.2519E-05 | 0.0295080000 |
| ENSG00000132639 | SNAP25 | -1.634759417 | 2.29E-05 | 0.016672004 | -1.269086667 | 1.14348E-06 | 0.0009490900 |
| ENSG00000120457 | KCNJ5 | 1.555450866 | 2.35E-05 | 0.016672004 | 2.0678 | 8.7722E-06 | 0.0047380000 |
| ENSG00000100427 | MLC1 | -2.039442496 | 2.36E-05 | 0.016672004 | -0.902633333 | 2.70324E-05 | 0.0072970000 |
| ENSG00000185298 | CCDC137 | 1.401613819 | 2.58E-05 | 0.017619146 | 0.912553333 | 6.26715E-05 | 0.0219781000 |
| ENSG00000248905 | FMN1 | 1.549049025 | 2.83E-05 | 0.018593513 | 1.28839 | 6.20525E-06 | 0.0042908000 |
| ENSG00000113494 | PRLR | 2.734223985 | 3.04E-05 | 0.018593513 | 1.46297 | 1.78237E-07 | 0.0000703150 |
| ENSG00000240720 | LRRD1 | 2.825615254 | 3.20E-05 | 0.018593513 | 1.12565 | 6.80384E-07 | 0.0005722400 |
| ENSG00000166780 | C16orf45 | -1.194817298 | 3.44E-05 | 0.018593513 | -0.895073333 | 0.00027231 | 0.0485770000 |
| ENSG00000167703 | SLC43A2 | 1.292517657 | 3.57E-05 | 0.018593513 | 1.11596 | 8.19071E-05 | 0.0355040000 |
| ENSG00000111716 | LDHB | -0.799627189 | 3.61E-05 | 0.018593513 | -1.186743333 | 4.2948E-05 | 0.0092671500 |
| ENSG00000207205 | RNVU1-15 | 1.909568009 | 3.69E-05 | 0.018593513 | 0.94929 | 4.15327E-05 | 0.0092671500 |
| ENSG00000248698 | LINC01085 | -2.191804211 | 4.30E-05 | 0.020422777 | -0.834036667 | 1.60851E-05 | 0.0065287000 |
| ENSG00000197696 | NMB | -1.541451733 | 4.37E-05 | 0.020422777 | -0.911856667 | 5.81331E-05 | 0.0181370000 |
| ENSG00000128512 | DOCK4 | 1.250285784 | 4.43E-05 | 0.020422777 | 1.32781 | 3.98041E-06 | 0.0018951500 |
| ENSG00000167202 | TBC1D2B | 1.072077477 | 4.71E-05 | 0.021257964 | 1.327683333 | 3.24597E-06 | 0.0018759200 |
| ENSG00000171914 | TLN2 | 1.651472399 | 4.87E-05 | 0.021495106 | 1.399796667 | 3.43852E-06 | 0.0018759200 |
| ENSG00000131398 | KCNC3 | 1.444337155 | 5.06E-05 | 0.021805171 | 0.823076667 | 9.36396E-05 | 0.0386387000 |
| ENSG00000125730 | C3 | 1.604441899 | 5.14E-05 | 0.021805171 | 1.400696667 | 5.64935E-07 | 0.0005533500 |
| ENSG00000259520 | LOC101928414 | -2.156931715 | 5.36E-05 | 0.022257808 | -1.14879 | 6.04352E-05 | 0.0183695000 |
| ENSG00000173442 | EHBP1L1 | 1.273799222 | 5.46E-05 | 0.022257808 | 0.886376667 | 0.000228341 | 0.0469763000 |
| ENSG00000198721 | ECI2 | -0.826613326 | 5.69E-05 | 0.022756868 | -1.087343333 | 7.58848E-05 | 0.0332790000 |
| ENSG00000175183 | CSRP2 | -1.636847515 | 5.91E-05 | 0.023183754 | -1.273663333 | 5.14497E-05 | 0.0130978650 |
| ENSG00000179583 | CIITA | 1.743934342 | 6.15E-05 | 0.023707749 | 0.901203333 | 3.9371E-05 | 0.0084630100 |
| ENSG00000121281 | ADCY7 | 1.493677358 | 6.93E-05 | 0.026227263 | 0.947493333 | 0.000137122 | 0.0411402000 |
| ENSG00000163431 | LMOD1 | -2.229855106 | 7.51E-05 | 0.027924212 | -1.28582 | 3.55266E-05 | 0.0075326000 |
| ENSG00000103365 | GGA2 | 0.712541251 | 7.72E-05 | 0.028188184 | 1.152413333 | 6.20613E-06 | 0.0042908000 |
| ENSG00000196782 | MAML3 | 1.185426755 | 7.94E-05 | 0.028188184 | 1.02581 | 9.3537E-06 | 0.0049454800 |
| ENSG00000113140 | SPARC | -1.412355087 | 7.98E-05 | 0.028188184 | -0.909836667 | 7.21047E-05 | 0.0284765000 |
| ENSG00000182628 | SKA2 | -0.883008408 | 8.36E-05 | 0.029042894 | -0.999726667 | 5.68E-06 | 0.0039177000 |
| ENSG00000105339 | DENND3 | 1.316706862 | 0.000102512 | 0.03342218 | 0.871916667 | 2.58E-05 | 0.0069736000 |
| ENSG00000176165 | FOXG1 | -1.63430048 | 0.000104276 | 0.033482033 | -1.229093333 | 1.45795E-05 | 0.0059290000 |
| ENSG00000134516 | DOCK2 | 1.199327646 | 0.000111491 | 0.034731883 | 0.82462 | 8.19121E-05 | 0.0355040000 |
| ENSG00000006047 | YBX2 | -2.313040195 | 0.000111563 | 0.034731883 | -1.576753333 | 4.60775E-07 | 0.0002001900 |
| ENSG00000110324 | IL10RA | 0.965774309 | 0.000113085 | 0.034731883 | 0.94937 | 7.5032E-05 | 0.0329340000 |
| ENSG00000185477 | GPRIN3 | 1.557687937 | 0.000138693 | 0.041396889 | 1.062763333 | 8.89579E-06 | 0.0047380000 |
| ENSG00000184156 | KCNQ3 | 1.223664442 | 0.000147503 | 0.043016539 | 0.654533333 | 0.000165039 | 0.0452860000 |
| ENSG00000186635 | ARAP1 | 0.988419235 | 0.000150209 | 0.043016539 | 1.38758 | 2.67328E-07 | 0.0000703150 |
| ENSG00000168918 | INPP5D | 1.327680814 | 0.000172388 | 0.048069 | 2.514093333 | 4.29526E-08 | 0.0000096326 |
| ENSG00000137841 | PLCB2 | 1.313362551 | 0.000178083 | 0.048695771 | 0.92301 | 2.32527E-05 | 0.0067862000 |
| ENSG00000071246 | VASH1 | 1.35675967 | 0.000179231 | 0.048695771 | 1.429313333 | 3.16209E-06 | 0.0018759200 |

Supplementary Table 8: Genes significantly differentially expressed on both RNAseq and gene expression array analysis.
