# Supplementary material for: Metabolism-based isolation of invasive glioblastoma cells with specific gene signatures and tumorigenic potential
Source: Neurooncol Adv. 2020 Jul 13;2(1):vdaa087. doi: 10.1093/noajnl/vdaa087 (PMC7462276; doi:10.1093/noajnl/vdaa087)
Supplement: vdaa087_suppl_Supplementary_Table_9 [file vdaa087_suppl_supplementary_table_9.docx]

| **Gene** | **Inv_FC** | **Inv_FDR** | **Rim_FC** | **Rim_FDR** | **Core_FC** | **Core_FDR** |
| --- | --- | --- | --- | --- | --- | --- |
| FOSB | 5.13 | 0.000701689 | 7.14 | 1.13E-05 | 6.05 | 0.000593441 |
| CXCL8 | 4.86 | 0.000701689 | 5.11 | 0.000163086 | 4.16 | 0.00458391 |
| TNFAIP3 | 3.91 | 0.001253541 | 4.74 | 0.000110793 | 3.92 | 0.002419517 |
| CCL20 | 3.79 | 0.015501353 | 4.05 | 0.007733978 | 3.52 | 0.029650218 |
| NR4A2 | 3.78 | 0.013942253 | 5.05 | 0.00134129 | 4.80 | 0.007289414 |
| DUSP2 | 3.70 | 0.00016664 | 3.31 | 0.000110793 | 3.31 | 0.0007893 |
| IL1B | 3.56 | 0.002542904 | 4.67 | 0.000110793 | 4.17 | 0.00139608 |
| IL1A | 3.47 | 0.014632766 | 4.13 | 0.004061998 | 3.41 | 0.024847055 |
| CXCL3 | 3.46 | 0.008228632 | 3.14 | 0.008299152 | 2.24 | 0.04855912 |
| CD83 | 3.44 | 0.008968541 | 4.84 | 0.000523469 | 4.10 | 0.007289414 |
| GPR183 | 3.15 | 0.008968541 | 4.48 | 0.000445878 | 4.72 | 0.00139608 |
| RNVU1-15 | 3.08 | 0.017741407 | 3.71 | 0.004788275 | 2.41 | 0.047836027 |
| CXCL2 | 3.00 | 0.017191138 | 2.78 | 0.018151255 | 2.58 | 0.036112855 |
| PPP1R15A | 2.97 | 0.002791498 | 2.65 | 0.003728249 | 1.67 | 0.045201464 |
| PTGER4 | 2.96 | 0.02565854 | 3.97 | 0.004061998 | 2.58 | 0.044733249 |
| EREG | 2.76 | 0.038723814 | 3.12 | 0.016591552 | 2.53 | 0.04855912 |
| KLF4 | 2.57 | 0.015501353 | 2.73 | 0.007733978 | 2.50 | 0.025537541 |
| BCL2A1 | 2.54 | 0.031081011 | 4.35 | 0.000745235 | 2.84 | 0.025537541 |
| C5AR1 | 2.50 | 0.040113066 | 4.08 | 0.00231421 | 4.30 | 0.00685996 |
| RNU11 | 2.49 | 0.009507481 | 2.76 | 0.003728249 | 2.56 | 0.016219292 |
| ATF3 | 2.46 | 0.029696813 | 3.00 | 0.007733978 | 2.49 | 0.032930926 |
| NR4A1 | 2.46 | 0.021973311 | 3.51 | 0.00231421 | 3.27 | 0.011121723 |
| KDM6B | 2.42 | 0.008968541 | 2.29 | 0.007733978 | 2.02 | 0.028878352 |
| NFKBIA | 2.39 | 0.008093017 | 2.93 | 0.000940469 | 3.08 | 0.003249621 |
| JUNB | 2.26 | 0.01652557 | 3.93 | 0.000246176 | 2.10 | 0.030168214 |
| FAM71A | 2.25 | 0.008093017 | 2.08 | 0.007665256 | 1.98 | 0.022938512 |
| HBEGF | 2.24 | 0.013702423 | 2.69 | 0.003077031 | 1.87 | 0.033381324 |
| ZNF331 | 2.23 | 0.017807092 | 2.32 | 0.010833026 | 2.56 | 0.018747883 |
| DUSP10 | 2.21 | 0.014254662 | 2.61 | 0.003728249 | 2.04 | 0.027615682 |
| ASTL | 2.19 | 0.003912993 | 2.17 | 0.002614703 | 1.94 | 0.015613875 |
| TRIB1 | 2.09 | 0.029696813 | 2.66 | 0.006571214 | 1.98 | 0.036329804 |
| KLF6 | 2.09 | 0.01964045 | 3.32 | 0.000745235 | 2.24 | 0.022938512 |
| LUCAT1 | 2.06 | 0.024586517 | 2.04 | 0.018366209 | 1.76 | 0.045165185 |
| DUSP1 | 2.04 | 0.039733985 | 3.80 | 0.000735283 | 2.83 | 0.016300793 |
| CSRNP1 | 2.02 | 0.017807092 | 2.50 | 0.004307393 | 2.03 | 0.025537541 |
| GRASP | 1.86 | 0.015501353 | 1.65 | 0.018719173 | 1.96 | 0.021917716 |
| STX11 | 1.80 | 0.029307714 | 2.52 | 0.003728249 | 2.73 | 0.007289414 |
| LSMEM1 | 1.80 | 0.009353755 | 1.47 | 0.017482121 | 1.69 | 0.022358809 |
| NFKB1 | 1.79 | 0.029696813 | 2.22 | 0.007468105 | 1.77 | 0.033813246 |
| CCNL1 | 1.78 | 0.014632766 | 2.07 | 0.004332008 | 1.58 | 0.030865731 |
| RNU12 | 1.75 | 0.038723814 | 2.04 | 0.013651262 | 2.46 | 0.015815858 |
| DNAJA1 | 1.73 | 0.014632766 | 1.64 | 0.013646224 | 1.40 | 0.037950464 |
| PPP1R15B | 1.69 | 0.002542904 | 1.77 | 0.000747692 | 1.59 | 0.007289414 |
| KYNU | 1.65 | 0.043167126 | 1.98 | 0.013646224 | 2.11 | 0.022938512 |
| TCHH | 1.58 | 0.048902684 | 1.91 | 0.014514481 | 2.00 | 0.025217703 |
| FOS | 1.54 | 0.017899529 | 3.85 | 1.13E-05 | 3.00 | 0.0007893 |
| HELB | 1.25 | 0.027304736 | 1.16 | 0.026884492 | 1.29 | 0.029650218 |
| ZFAND5 | 1.23 | 0.00979609 | 1.33 | 0.004332008 | 1.51 | 0.007289414 |
| TEX14 | 1.17 | 0.046952381 | 1.49 | 0.010833026 | 1.39 | 0.028878352 |
| FNIP2 | 1.16 | 0.047880661 | 1.24 | 0.025581184 | 1.37 | 0.029650218 |
|  |  |  |  |  |  |  |
| ANGPT2 | -3.51 | 0.008228632 | -2.64 | 0.020318437 | -2.30 | 0.047380695 |
| MYO1B | -3.21 | 0.008093017 | -2.09 | 0.040738991 | -2.59 | 0.028681857 |
| BGN | -3.20 | 0.008968541 | -2.42 | 0.021770718 | -2.54 | 0.030865731 |
| IGFBP7 | -3.05 | 0.002542904 | -2.06 | 0.013651262 | -1.68 | 0.045165185 |
| ABCC9 | -2.88 | 0.002542904 | -1.85 | 0.017448728 | -1.54 | 0.04693121 |
| NID1 | -2.80 | 0.019971967 | -2.26 | 0.04120126 | -2.57 | 0.034767225 |
| SNORD114-14 | -2.79 | 0.038723814 | -4.02 | 0.004416716 | -3.53 | 0.022938512 |
| COL4A2 | -2.75 | 0.005027712 | -2.15 | 0.011658816 | -2.37 | 0.019140454 |
| COL4A1 | -2.63 | 0.014632766 | -1.86 | 0.049423144 | -2.06 | 0.041528685 |
| DDR2 | -2.56 | 0.004426861 | -2.42 | 0.003728249 | -3.45 | 0.00139608 |
| LAMA4 | -2.18 | 0.014632766 | -1.90 | 0.018979168 | -2.82 | 0.007752213 |
| MTSS1L | -2.15 | 0.022710918 | -2.37 | 0.010375726 | -1.92 | 0.037964492 |
| HSPG2 | -2.12 | 0.014254662 | -1.72 | 0.026036588 | -1.99 | 0.026254072 |
| LOXL2 | -2.11 | 0.032469345 | -1.80 | 0.048581693 | -3.44 | 0.00685996 |
| LAMB2 | -2.10 | 0.008093017 | -2.00 | 0.006598479 | -2.82 | 0.002561185 |
| WLS | -1.96 | 0.013942253 | -2.07 | 0.007468105 | -1.88 | 0.024593498 |
| ECE1 | -1.96 | 0.029696813 | -1.80 | 0.03037209 | -2.21 | 0.025217703 |
| MKL2 | -1.90 | 0.008093017 | -1.68 | 0.008423764 | -1.82 | 0.016219292 |
| PTPRG | -1.89 | 0.019971967 | -1.94 | 0.013144469 | -2.92 | 0.00458391 |
| LEF1 | -1.85 | 0.013702423 | -1.78 | 0.010239848 | -1.55 | 0.033600881 |
| PLEKHA4 | -1.76 | 0.008968541 | -1.37 | 0.01951732 | -1.69 | 0.019990242 |
| EPAS1 | -1.71 | 0.031081011 | -1.67 | 0.023215891 | -1.68 | 0.035396305 |
| MT1M | -1.66 | 0.019045515 | -1.69 | 0.013144469 | -1.32 | 0.048365474 |
| PAM | -1.60 | 0.031944484 | -1.71 | 0.017448728 | -2.30 | 0.011121723 |
| NPAS2 | -1.48 | 0.019054745 | -1.64 | 0.008121715 | -1.95 | 0.010308806 |
| GDF11 | -1.39 | 0.043167126 | -1.79 | 0.009498522 | -2.05 | 0.014686261 |
| FNDC4 | -1.37 | 0.0389852 | -1.47 | 0.020017028 | -1.91 | 0.016219292 |
| SLC1A1 | -1.26 | 0.037722208 | -1.35 | 0.01951732 | -1.19 | 0.044733249 |
| CX3CL1 | -1.05 | 0.04339562 | -1.34 | 0.010305096 | -1.08 | 0.03835822 |

Supplementary Table 9: List of genes significantly up (positive FC) or down (negative FC) regulated between 5ALA positive cells and Invasive (Inv FC), rim (rim FC) or core (core FC) regions with associated p value after FDR correction
