# Supplementary figures and images for: Metabolism-based isolation of invasive glioblastoma cells with specific gene signatures and tumorigenic potential
Source: Neurooncol Adv. 2020 Jul 13;2(1):vdaa087. doi: 10.1093/noajnl/vdaa087 (PMC7462276; doi:10.1093/noajnl/vdaa087)

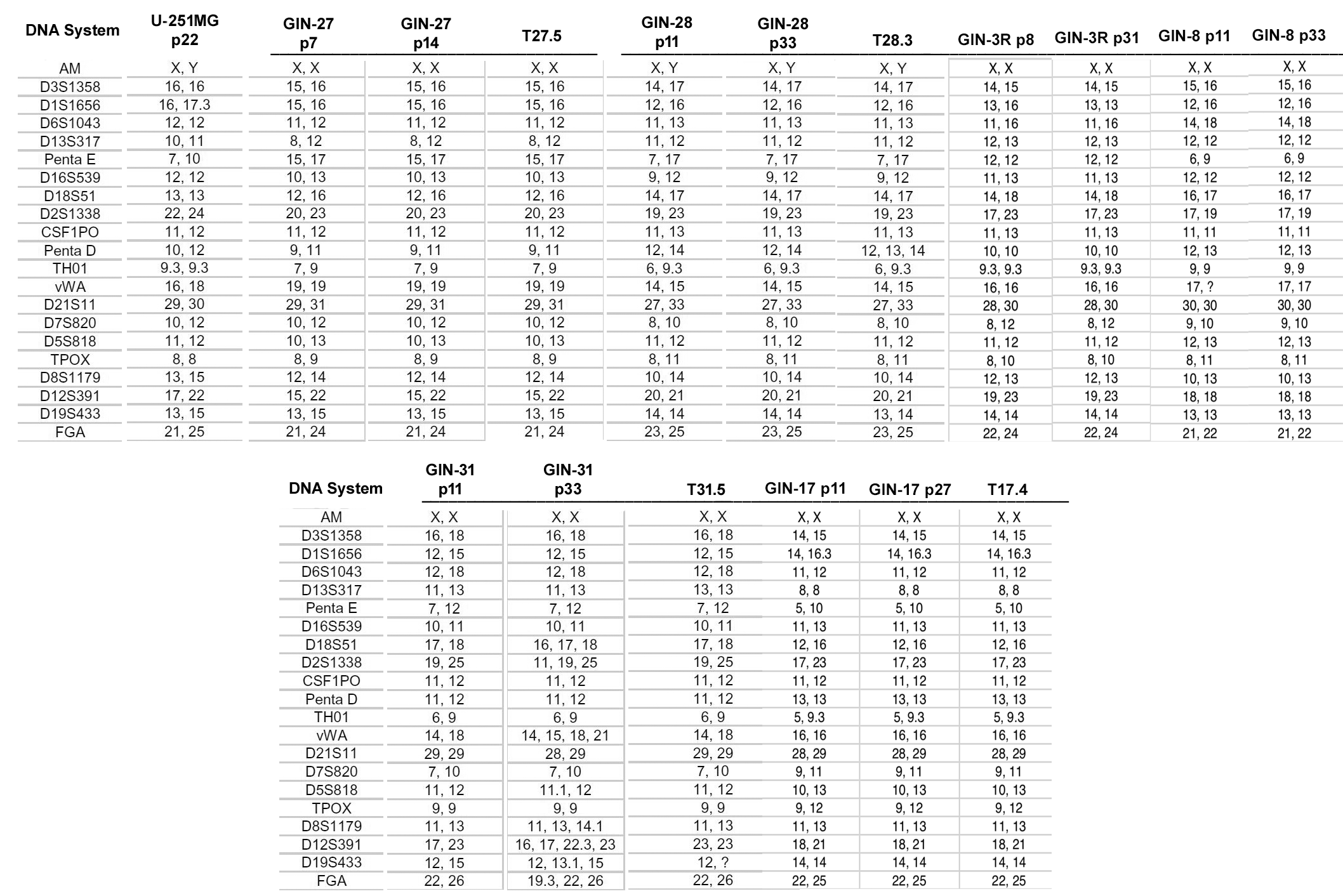

Supplement: vdaa087_suppl_Supplementary_Figure_1 [file vdaa087_suppl_supplementary_figure_1.png]

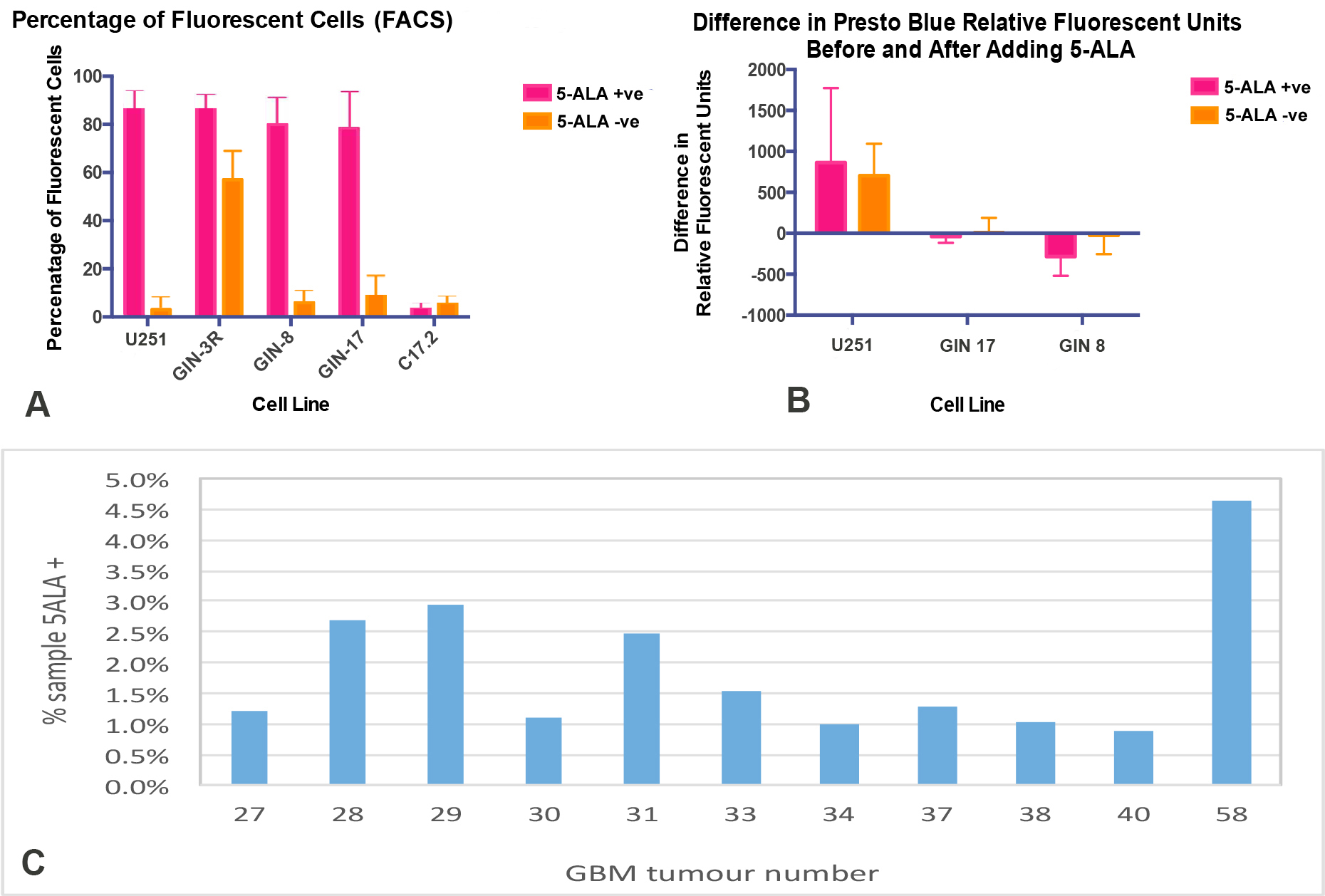

Supplement: vdaa087_suppl_Supplementary_Figure_2 [file vdaa087_suppl_supplementary_figure_2.png]

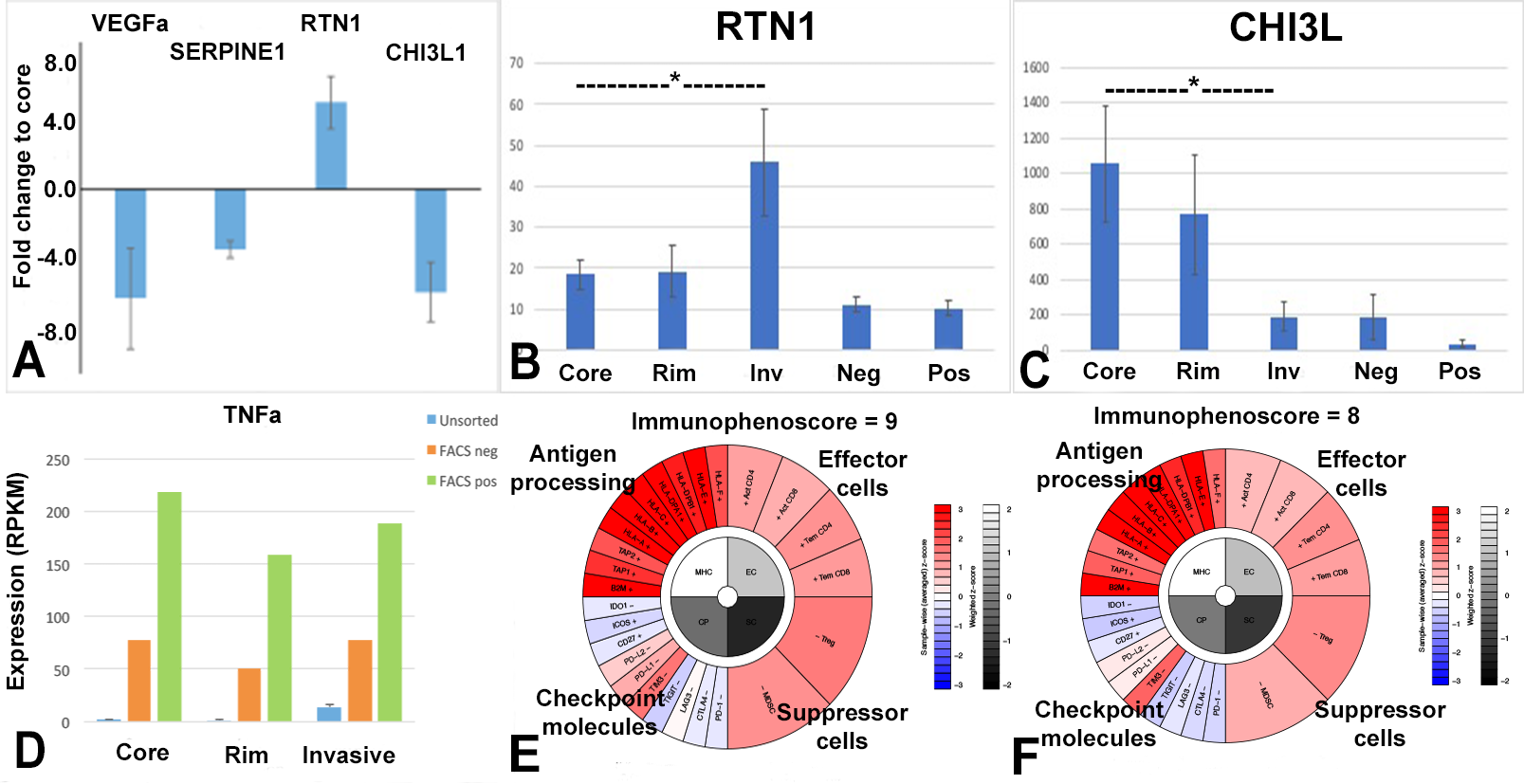

Supplement: vdaa087_suppl_Supplementary_Figure_3 [file vdaa087_suppl_supplementary_figure_3.png]

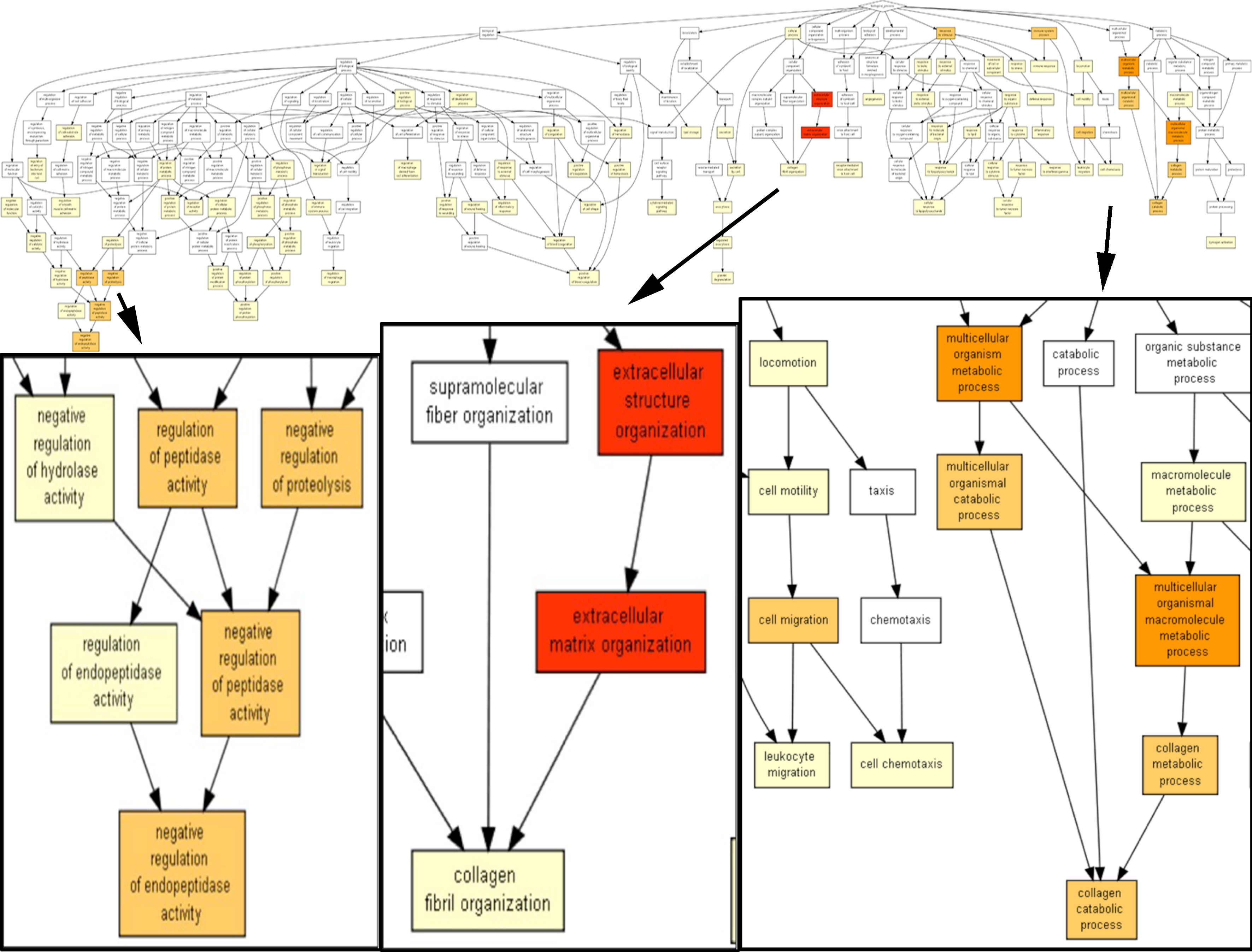

Supplement: vdaa087_suppl_Supplementary_Figure_4 [file vdaa087_suppl_supplementary_figure_4.png]

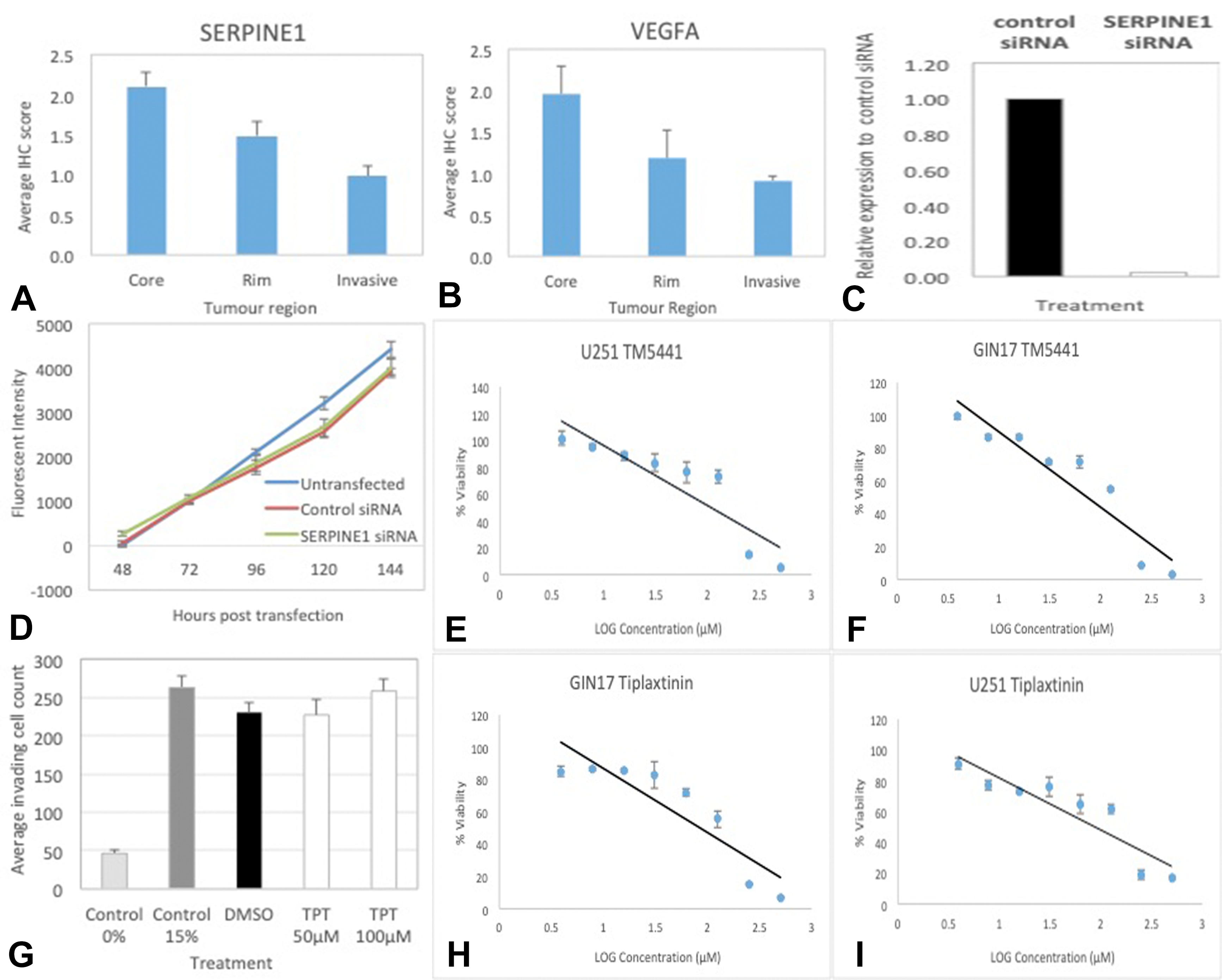

Supplement: vdaa087_suppl_Supplementary_Figure_5 [file vdaa087_suppl_supplementary_figure_5.png]

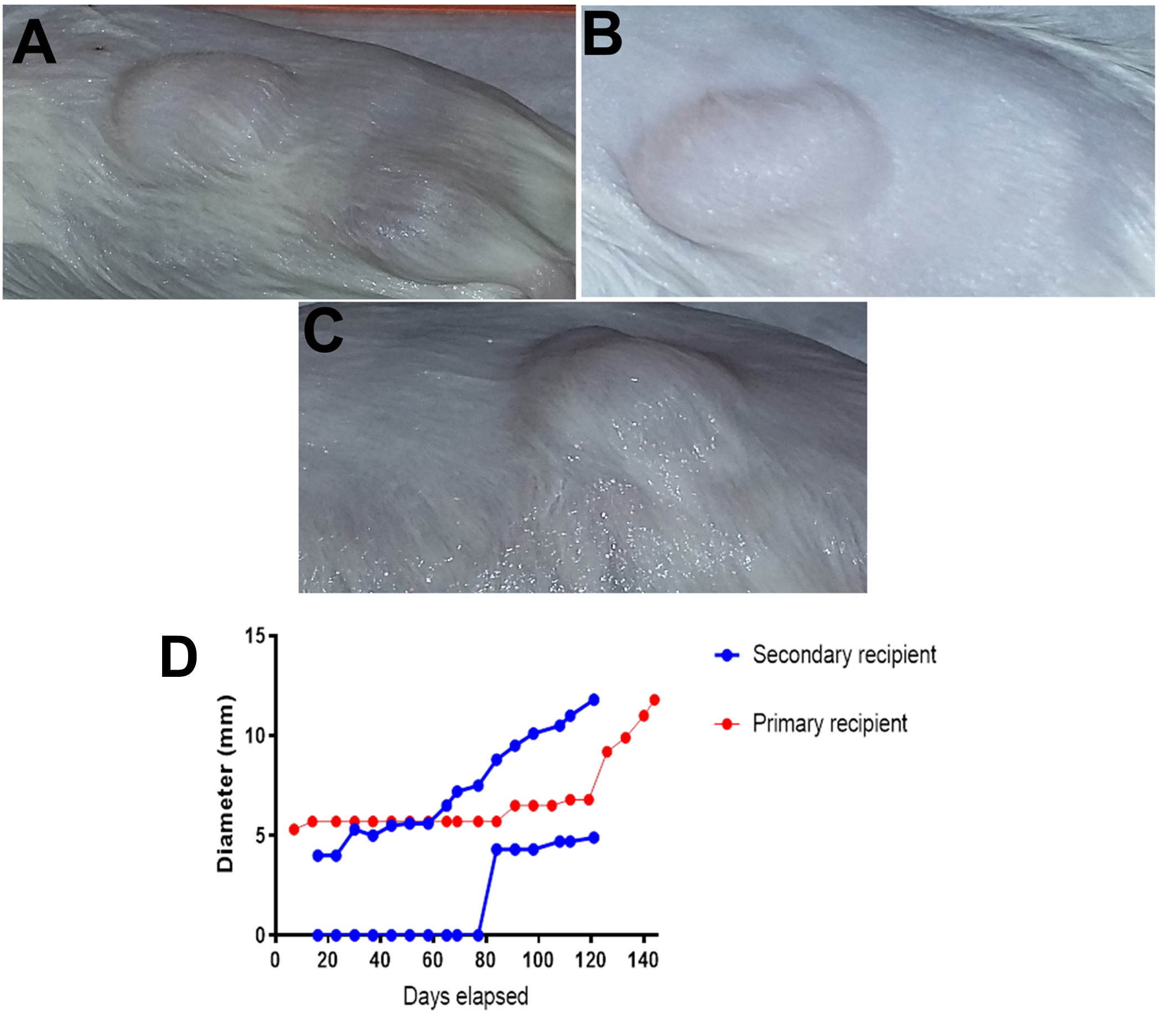

Supplement: vdaa087_suppl_Supplementary_Figure_6 [file vdaa087_suppl_supplementary_figure_6.png]
