# Supplementary material for: Metabolism-based isolation of invasive glioblastoma cells with specific gene signatures and tumorigenic potential
Source: Neurooncol Adv. 2020 Jul 13;2(1):vdaa087. doi: 10.1093/noajnl/vdaa087 (PMC7462276; doi:10.1093/noajnl/vdaa087)
Supplement: vdaa087_suppl_Supplementary_Figure_Legends [file vdaa087_suppl_supplementary_figure_legends.docx]

**Supplementary Figure Legends**

Supplementary Figure 1

Short tandem repeat (STR) genotyping data for U251 (match to ATCC control) and all the primary lines used in the study. All primary lines displayed genetic stability over several passages and compare with the corresponding tumor tissue region from which they were derived (T17.4, T27.5, T28.3 and T31.5).

Supplementary Figure 2

A – Percentage of fluorescent cells on flow cytometry for U251, GIN3R, GIN8 and GIN17 primary lines and the C17.2 neural stem cell line B – Metabolic viability Presto blue assay on U251, GIN17 and GIN8 cell lines with and without 5ALA C – Percentage of each individual GBM tumor sample sorting as positive on FACS based on 5ALA fluorescence.

Supplementary Figure 3

A - rtPCR validation for 4 selected genes demonstrating fold changes in invasive tissue comparable to the RNAseq data (shown for VEGFa in figure 3E, for SERPINE 1 in figure 4G and for RTN1 and CHI3L in this figure B and C) . B – RNAseq expression levels for gene RTN1 (asterix indicating significant differential expression between core and invasive tumor cells, p<0.05) C – RNAseq expression levels of CHI3L (asterix indicating significant differential expression between core and invasive tumor cells, p<0.05) D - RNAseq expression profiles comparing unsorted cells with 5ALA positive and negative cells after FACS across tumor core, rim and invasive regions showing FACS induced elevation of *TNFα*, particularly in 5ALA positive cells. E&F - Immunophenoscore profiles for matched unsorted tumor core (E) and invasive (F) regions with high expression of antigen processing and relatively low expression of most checkpoint molecules.

Supplementary Figure 4

Pathway diagram illustrating foci of abnormal pathway activity in peptidase activity, extracellular organization and cell migration and metabolism, with red/orange coloring indicating higher levels of gene involvement in a particular pathway.

Supplementary Figure 5

A&B – Mean score for SERPINE1 (A) and VEGFA (B) immunohistochemistry for core, rim and invasive tumor regions. C – Expression levels of *SERPINE1* before and after siRNA mediated knockdown in U251. D – Viability levels measured by PrestoBlue fluorescent assay for U251 comparing untransfected (blue line), scrambled siRNA control (red line) and targeted siRNA knockdown of *SERPINE1* (green line). E&F – Viability of U251 (E) and GIN17 primary (F) in response to TM5441. G – Invasion of U251 cells in transwell collagen invasion assay in response to control with 0% or 15% serum compared to DMSO alone or Tiplaxtinin at 50uM or 100uM. H&I – Viability of GIN17 and U251 cells in response to varying concentration of Tiplaxtinin.

Supplementary Figure 6

Visible tumor mass from patient GBM subcutaneous xenograft (GBM3) generated from freshly resected, (A) primary unsorted tumor core, (B) primary unsorted invasive margin and (C) one of two 5ALA/FACS positive cell subcutaneous serial injections from the primary xenograft, that resulted in tumor uptake in a secondary recipient. (D) Serial transplant of 5ALA/FACS positive xenograft tumor into two secondary Rag2G recipients at day 145 (post tumor implant into primary recipients), with one injection (blue line; top) resulting in an increased rate (approximately double) of tumor uptake (69 days to reach a tumor diameter of 7.2 mm, relative to 119 days for a 6.8 mm diameter tumor in the primary recipient (red line)). The second serial injection of 5ALA/FACS positive tumor cells into a secondary recipient (blue line; bottom) failed to generate a subcutaneous xenograft until day 80 and never reached greater than 5mm diameter.
