# Supplementary material for: Metabolism-based isolation of invasive glioblastoma cells with specific gene signatures and tumorigenic potential
Source: Neurooncol Adv. 2020 Jul 13;2(1):vdaa087. doi: 10.1093/noajnl/vdaa087 (PMC7462276; doi:10.1093/noajnl/vdaa087)
Supplement: vdaa087_suppl_Supplementary_Methods [file vdaa087_suppl_supplementary_methods.docx]

Tumor Dissociation

Tumor dissociation was as per Hussein et al. ^18^. RNA was extracted using mirVANA^TM^ miRNA Isolation Kit (Ambion; Life Technologies, Paisley, UK) according to the manufacturer’s instructions. RNA samples were assessed for purity and quantity using Qubit (Thermo Fisher) and 2100 Bioanalyser (Agilent).

Cell culture

1 ml 20 mM 5-ALA (Sigma-Aldrich, St. Louis, Missouri, USA) was diluted in 9 ml growth medium to obtain a 1:10 solution of 5ALA at a working concentration of 2 mM. These solutions were protected from light. The growth medium was removed from each flask/plate and the cells were washed with HBSS. 2 mM 5ALA solution was then added to each of the 5ALA positive wells/flasks, and the same amount of fresh growth medium with 15% FBS added to the 5ALA negative wells/flasks. The cells were then incubated for 2 hours at 37 °C in 5 % CO_2_ air-humidified atmosphere.

PrestoBlue® (Invitrogen by Life Technologies, Carlsbad, California, USA) is a cell permeable, blue resazurin-based solution that is converted into an intensely red-fluorescent dye when in the presence of a metabolic substrate within the mitochondria of cells. After 2 hours incubation, the 5ALA was removed and the PrestoBlue®assay repeated. Exposure times used for bright-field were 4 ms, while the exposure for imaging 5ALA fluorescence was 30 s using a DsRed filter (wavelength 587-610nm). Cells were cultured to 60-80% confluency within 48 hours with all flasks incubated at 37 °C in 5 % CO_2_ air-humidified atmosphere.

At this stage, C17.2 flasks were replenished with differentiation medium. The growth medium was pipetted off, cells were washed with HBSS and the differentiation medium added and incubated for 24 hours at 37 °C in 5 % CO_2_ air-humidified atmosphere (until used for flow cytometry). 500μl of 5ALA solution was added to each 5ALA positive flask containing 5 ml of growth medium. Following incubation for 2 hours, all medium was removed flasks washed with 1ml HBSS. Trypsin-dissociated pellets were resuspended in 15 % Phenol-red free DMEM (3 ml for U251 and 1 ml for GIN-3R, GIN-8, GIN17 and C17.2) before cell counting.

The cell number and concentration required for flow cytometry was a V_f_ of 1 ml and C_f_ of 200000cells/ml. The MoFlo XDP high-speed cell sorter (Beckman Coulter, Brea, California, USA) was used, using the violet laser with the FL11 channel (λex 405nm, λem filter 615/20 and λem bandwidth 605-625nm). These parameters were chosen to match known 5ALA excitation and emission bands.

Real-Time PCR

cDNA was synthesized from 0.5 µg TURBO^TM^ DNAse (Life Technologies) treated RNA for each region using RevertAid reverse transcriptase (Thermo Scientific). The synthesized cDNA of each sample was mixed with iQ^TM^ SYBR® Green Supermix 2x (Bio-Rad), forward and reverse primers (Supplementary Methods Table 1) and high-quality nuclease free water. All RT-PCR reactions were run on the CFX96^TM^ RT-PCR detection system (Bio-Rad). The cycle conditions for RT-PCR were 95 °C for 3 minutes, followed by 40 cycles of 95 °C for 20 seconds, 60 °C for 30 seconds and 72 °C for 30 seconds. Relative quantification was determined using the Pfaffl equation with GAPDH used as the reference gene.

Immunohistochemistry

Tissue Microarrays (TMAs) were created and samples separated in the TMA by tumor region and by patient. After de-paraffinization using an alcohol series, samples were placed in a pressure cooker with sodium citrate buffer (pH 6) for 8 minutes. Slides were then blocked with 20 % goat serum for 20 minutes and peroxidase blocking solution for 5 minutes. Primary antibodies were applied diluted in antibody diluent (DAKO, Ely, Cambridgeshire, UK), 1:200 dilution for SERPINE1 (Abcam, ab66705) and 1:25 dilution for VEGFA (DAKO, M7273) and incubated for 2 hours at room temperature. Sections were washed with PBS for 5 minutes and the secondary antibody (DAKO) was applied to cover the specimen and incubated at 37 °C for 30 minutes. Following another PBS wash, DAB solution was applied and incubated for 5 minutes. Slides were counterstained in Harris Haematoxylin for 10 seconds before dehydrating using an alcohol series and xylene. Each region was scored for intensity and proportion of positively stained cells and averaged across all the patients to generate a weighted score for each region.

Transcriptomics and Biostatistics

Libraries were prepared using the NEBNext Poly(A) mRNA Magnetic Isolation Module (NEB: E7490), the NEBNext Ultra Directional Library Kit for Illumina (NEB: E7420) and the NEBNext Multiplex Oligos for Illumina (Index Primers Set 1) (NEB: E7335L). For samples with total RNA concentrations over 10 ng/µl, 0.5 µg of total RNA was used for library preparation. To ensure sufficient library concentrations were obtained from each sample, 14 cycles of amplification were used during the PCR Library enrichment step.

Finished libraries were quantified using the Qubit dsDNA HS kit (Invitrogen: Q32854). Library concentrations and fragment size distributions were also analyzed using the Agilent Bioanalyzer High Sensitivity DNA Kit (Agilent: 5067-4626).

Libraries were normalized to 2 nM and pooled in equimolar amounts. The Kapa Library Quantification Kit, for Illumina Platforms (KAPA Biosystems: KK4824), was used for precise quantification of the library pool. The library pool was denatured and diluted to 1.6 pM, spiked with 1 % PhiX (1.8 pM) and sequenced on the Illumina NextSeq 500, using the NextSeq 500/550 High Output v2 Kit (150 cycles) (Illumina: FC-404-2005), to generate a minimum of 70 million pairs of 75-bp paired-end reads per sample (raw data deposited at ArrayExpress, accession number E-MTAB-8743).

The trimming pipeline was used to filter reads with low sequencing score as well as reads aligned to adaptor sequences. First, raw reads were trimmed against adaptors, and then reads were quality trimmed by Skewer ^19^. In order to reduce the background noise of human tRNAs and rRNAs, trimmed reads were aligned and filtered against both components first by bwa mapping tools. Then reads which passed the filters were mapped onto the reference genome in the context of known gene exon coordinates by the Hisat2 mapping tool ^20^ with genome reference hg38 and annotation source Ensembl.

'Primary' mapped reads were recorded as the best mapping position of each read, regardless of whether they mapped uniquely. Read alignments, both primary and unique, were filtered further according to their mapping quality score (MAPQ).

Read counts for each gene were calculated using 'htseq-count'. (<http://www-huber.embl.de/users/anders/HTSeq/doc/count.html>.) The program determines the number of uniquely and correctly aligned reads per gene. The RPKM is a normalized read count (stranded/sense reads) for a given gene. The read count of the exon-space of a gene was normalized against the total number of mapped reads (with rRNA excluded and reads not uniquely aligned and below MAPQ20 included) in that particular alignment file, and against the total length of the exon-space of a gene.

Principal component analysis (PCA) was used as an orthogonal transformation to convert a set of observations of possibly correlated variables into a set of values of linearly uncorrelated variables called principal components. In analysis, PCA was used to confirm that the biological replicates were sufficiently close to each other. DESeq was used to detect the differentially expressed genes for each comparison, and a p-value < 0.05 and fold-change > 2 was used as the DESeq thresholds. Genes were filtered out with an average rpkm < 1. Pathway analysis was conducted using GO ontology and GOrilla software.

Genome-wide gene expression microarray analysis samples were run on the Affymetrix Gene 2.1 ST array after RNA extraction and preparation. Quality control was conducted on a bioanalyzer with RNA integrity number (RIN) of 7 or greater. Analysis of normalized data was conducted using linear models.

siRNA-mediated knockdown

U251 and GIN-17 cells were seeded at 100,000 cells/well in 6 well plate and allowed to adhere overnight. 25 nM of SMARTpool: ON-TARGETplus SERPINE1 (Dharmacon) or ON-TARGETplus Non-targeting Pool (Dharmacon) siRNA was transfected using DharmaFECT 1 transfection reagent (Dharmacon) for 24 to 48 hours using a protocol recommended by Dharmacon. To determine silencing of SERPINE1, RNA was harvested 24 hours after transfection and SERPINE1 mRNA levels were evaluated using real-time PCR.

*In vitro* invasion assay

24 well ThinCert^TM^ cell culture inserts (Greiner) were coated with 10 µg of collagen IV (Cultrex, Trevigen). U251 and GIN-17 cells were seeded at 15,000 cells/insert in 200 μl 1% FBS DMEM media. Cells invaded towards 700 μl 10% FBS DMEM media in each well for 24 hours in a cell culture incubator at 37 °C 5 % CO_2_. Invasion towards 1% FBS DMEM media was used as a control. After 24 hours medium was removed from each insert and cells fixed with 700 µl of ice cold 100% methanol for 1 hour at room temperature. To stain cells, inserts were incubated in 600 µl of 0.2% crystal violet for 15 minutes at room temperature. Inserts were then washed in distilled water and left to dry overnight. Cells on the underneath of the insert were counted for 4 x 200 images per insert and averaged. TM5441 (5-Chloro-2-[[2-[2-[[3-(3-furanyl)phenyl]amino]-2oxoethoxy]acetyl]amino]benzoic acid) (Tocris) was utilized as a specific *SERPINE1* inhibitor as described in the literature^21^. Tiplaxtinin (R&D) was also utilized as a SERPINE1 inhibitor in the same manner.

*In vivo* tumorigenicity

Male Rag2/Il2rg (RAG) immunodeficient mice (10-12 weeks old) were sourced from our in-house colony. Mice were maintained in Individually Ventilated Cages (IVCs) (Tecniplast UK) within a barriered unit illuminated by fluorescent lights set to give a 12 hour light-dark cycle (on 07.00, off 19.00), as recommended in the United Kingdom Home Office Animals (Scientific Procedures) Act 1986. The room was air-conditioned by a system designed to maintain an air temperature range of 21 ± 2ºC and a humidity of 55% + 10%. Mice were housed in social groups during the procedure with irradiated bedding and provided with autoclaved nesting materials and environmental enrichment. Sterile irradiated 5V5R rodent diet (IPS Ltd, UK) and irradiated water (Baxter) was offered ad libitum.

Freshly resected tumor was dissociated and sorted by 5ALA-induced fluorescence as described. Cells were implanted directly as mixed core populations, mixed invasive margin populations or 5ALA FACS positive invasive margin cells *in vivo* (i.e. without any intervening period of *in vitro* propagation). Single cell suspensions were implanted into the left flank of primary recipient male RAG mice, using 1 million cells per implant (n=3 except 5ALA positive which was n=2) for each sampled tumor region. 5ALA FACS positive tumors were subsequently serially transplanted into flanks of secondary recipient male RAG mice (n=2).

Tumors were measured weekly using Vernier calipers and the volumes calculated using the formula *V*=*ab*^2^
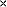
*
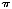
*/6, where *a* is the length and *b* is the width. Mice were also weighed weekly and checked daily by an experienced technician in full compliance with legal and ethical guidelines as detailed in the UK Home Office Animals (Scientific Procedures) Act 1986 (Home Office PPL no. P435A9CF8). NCRI guidelines for the welfare and use of animals in cancer research, LASA good practice guidelines and FELASA working group on pain and distress guidelines were also followed.
